# The biogeography of giant viruses and interactions with Eukaryotes across European lakes

**DOI:** 10.1038/s41467-026-77765-w

**Published:** 2026-09-23

**Authors:** Olesya V. Kolmakova, Frederik Schulz, Yumary M. Vasquez, Jeremy A. Fonvielle, Lucas P. P. Braga, Simon Roux, Tanja Woyke, Andrew J. Tanentzap

**Affiliations:** 1 Ecosystems and Global Change Group, Department of Plant Sciences, University of Cambridge, Cambridge, CB2 3EA UK; 2 United States Department of Energy Joint Genome Institute, Lawrence Berkeley National Laboratory, Berkeley, CA USA; 3 University of California Merced, Life and Environmental Sciences, Merced, CA USA; 4 Ecosystems and Global Change Group, School of Environment, Trent University, Peterborough, ON K9L 0G2 Canada

**Keywords:** Microbial ecology, Climate-change ecology, Freshwater ecology, Biogeography

## Abstract

Advances in genomics have revealed a diversity of nucleocytoplasmic large DNA viruses (phylum Nucleocytoviricota) that infect eukaryotes, but their ecology beyond the oceans remains poorly understood. We recover 208 Nucleocytoviricota genomes, including 205 putative new species and 33 novel virus-host linkages by sampling 87 lakes spanning 30° of latitude from southern Italy to northern Norway. The relative abundance of Nucleocytoviricota nearly triples, on average, from northern to southern Europe, with functional genes related to autotrophic energy acquisition, stress responses, and cell death predominant at lower latitudes. These shifts are attributable to differences in light and resources, with Nucleocytoviricota hotspots in deep lakes with aromatic organic matter and diverse eukaryote communities. Increasing Nucleocytoviricota abundance and richness are associated with several-fold reductions in gross primary production. Our results highlight that Nucleocytoviricota are ecologically relevant in lakes and that understanding their roles will be essential for predicting aquatic food webs under environmental change.

## Abstract (web summary)

Large and giant DNA viruses are widespread in nature but their ecology remains poorly understood in freshwater ecosystems. This study finds that giant viruses are abundant in warmer waters where they are linked to lower primary production.

## Introduction

Recent advances in metagenomics have facilitated the discovery of a tremendous diversity and widespread distribution of nucleocytoplasmic large and giant DNA viruses in the phylum Nucleocytoviricota^1^. Nucleocytoviricota are known to infect eukaryotes exclusively and include viruses with megabase-sized genomes and a particle size that rivals that of bacteria^1^. However, much of our understanding of Nucleocytoviricota distributions and their interactions with their hosts comes from marine environments^2–6^. In freshwater lakes and rivers, which deliver vital ecosystem services, the composition of Nucleocytoviricota and their interactions with host taxa remain largely unknown^7,8^. Although more than 1000 Nucleocytoviricota metagenome-assembled genomes (MAGs) have been described from freshwaters^6^, their ecological roles remain understudied.

The limited understanding of the ecology of Nucleocytoviricota in freshwater systems hinders our ability to predict their responses to global change and their broader implications for food webs and biogeochemical cycling. Changes in the distributions of Nucleocytoviricota in response to global change drivers, such as warming temperatures and rising nutrient export from surrounding land use, can reorganise large-scale carbon and energy flows through eukaryotes across all trophic levels^9–11^. For example, in the oceans, Nucleocytoviricota are predicted to accelerate biological carbon export from the productive surface layer to deep layers by promoting cell death and aggregation of their hosts^5^. Consequently, Nucleocytoviricota contribute to the export of between 4 and 12 Pg C year^−1^ into the mesopelagic zone^12,13^, which is comparable in magnitude to some of the largest fluxes in the global carbon cycle^14^. Nucleocytoviricota can also engage in other trophic interactions beyond directly altering the population dynamics of their hosts. For example, unlike other viruses, large Nucleocytoviricota particles are sometimes directly preyed upon by heterotrophic eukaryotes, such as ciliates^15^. Protists can also preferentially consume phytoplankton cells infected by Nucleocytoviricota because chemicals released by lysed algae attract grazers^16^. Nucleocytoviricota also engage in non-trophic interactions with eukaryotes. For example, the *Algavirales Gephyrocapsa huxleyi* virus uses zooplankton as a dispersal vector to reach its algal hosts^17^. By infecting and lysing these hosts, the virus reduces algal availability and thus indirectly affects zooplankton without a direct trophic interaction.

Nucleocytoviricota also play an important role in reprogramming eukaryotic host genomes and modifying their metabolism through the many functional genes in their large genomes^1^. Nucleocytoviricota can reprogram their eukaryotic hosts through horizontal gene transfer (HGT), where viral genes are integrated into the host genome to facilitate viral replication and manipulate host cellular processes^18^. Nucleocytoviricota also carry auxiliary metabolic genes (AMGs), which encode proteins that modify host metabolism to create a more favourable environment for viral propagation^19^. Over longer timescales, these mechanisms, alongside HGT from eukaryote hosts into Nucleocytoviricota genomes^20^, can facilitate coevolution between viruses and their hosts that further reshapes host genomes and metabolism. This coevolution is particularly relevant in rapidly changing environments, as it can foster reciprocal adaptations that allow host-virus systems to remain functional as external conditions shift. Understanding the interactions between Nucleocytoviricota and their hosts at both genetic and ecosystem levels is therefore essential for predicting how freshwater ecosystems will respond to global change.

Here, we investigated how freshwater Nucleocytoviricota respond along gradients of global change drivers and influence wider ecological dynamics. We surveyed Nucleocytoviricota and their putative host interactions in the surface waters of 87 lakes spanning 30° of latitude from southern Italy to northern Norway (Supplementary Data 1). Our latitudinal sampling acted as a space-for-time substitution for future climate warming by comparing Nucleocytoviricota across locations that experienced different mean annual temperatures and surrounding land uses, while statistically controlling for variation in other environmental parameters. Using shotgun metagenomic sequencing, we obtained a mean of 58 million (standard deviation: 24 million) reads per lake (Supplementary Data 1) and identified 208 Nucleocytoviricota MAGs whose co-occurrence we matched with 174 eukaryotic metagenomic contigs containing an 18S rRNA gene to identify putative virus-host linkages. We then analysed the biogeographic distribution of Nucleocytoviricota MAGs and their functional genes and tested how these varied across our large-scale environmental gradients. Consistent with the latitudinal gradient hypothesis^21^, we predicted higher Nucleocytoviricota abundance and diversity at lower latitudes where warmer temperatures and more nutrient-rich waters increase host availability and the efficiency of viral AMGs^22,23^. Given the importance of algae as hosts of Nucleocytoviricota^6^, we hypothesised that Nucleocytoviricota infections of algae, in particular, will become more prevalent at warmer temperatures. Alongside an increased occurrence of algae genome modifications via HGT, we expected ecosystem-level measures, such as primary productivity, to vary with the richness and abundance of Nucleocytoviricota. We also predicted that other environmental conditions, such as underwater irradiance, would influence algal distributions and, consequently, Nucleocytoviricota richness and abundance. By understanding these biogeographic patterns, our study helps to predict how viral communities may respond to global change and impact freshwaters.

## Results

### Nucleocytoviricota and their predicted hosts in European lakes

**Fig. 1:**
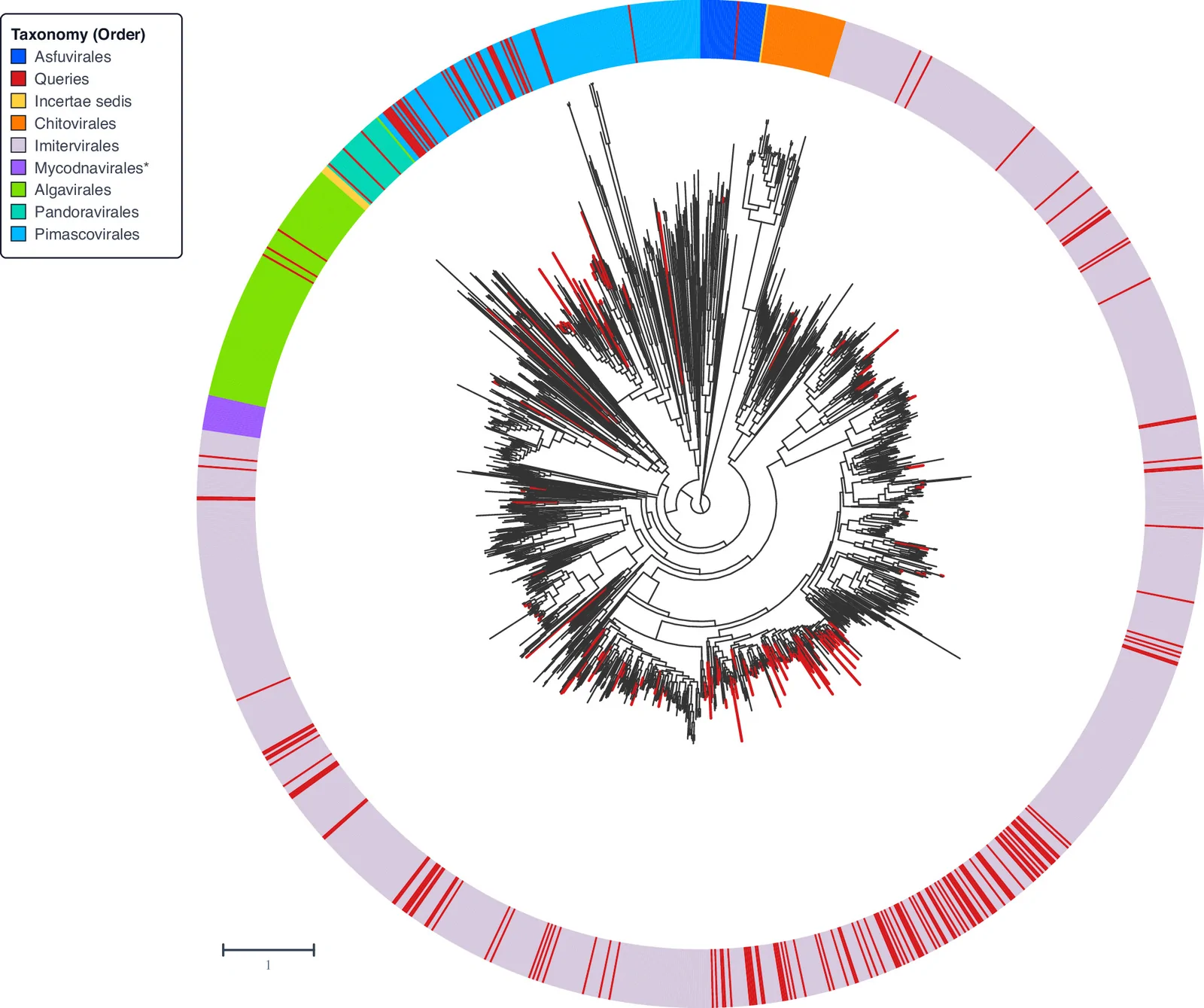
Maximum-likelihood phylogeny of MAGs identified in this study placed within the Nucleocytoviricota. The tree was inferred from a concatenated amino acid supermatrix of seven giant virus orthologous group markers using IQ-TREE v3.1.1 with the Q.pfam+F + R10 substitution model. The final supermatrix comprised 7487 sites across 206 genomes identified in this study and 1383 reference genomes. The tree was rooted at Pokkesviricetes (Asfuvirales + Chitovirales) and the scale bar represents the number of substitutions per site. Red branches are query-associated MAGs discovered in this study. Clades that contain queries are highlighted in grey and labeled with the respective Family name. *indicates Imitervirales clade that has recently been proposed to represent a new Order called Mycidnavirales^76^.

Across all 87 lakes, we retrieved 208 Nucleocytoviricota MAGs, of which >95% had a high confidence match to the phylum Nucleocytoviricota and all but three were completely novel species (Supplementary Data 2, 3). All but 3 MAGs were >100 kbp and within the range of published Nucleocytoviricota genome sizes^24^, further emphasising their high quality (Supplementary Data 4). These results were robust to potential under-sampling of MAGs because of unassembled genomes as the richness of the Nucleocytoviricota marker gene DNA polymerase B (PolB) strongly correlated with MAG richness across all lakes (Spearman rank correlation, ρ = 0.76, p-value < 0.001). To place the novel MAGs in an evolutionary and taxonomic context, we then constructed a phylogenetic tree including all 208 MAGs retrieved in this study alongside 1383 representative reference genomes, enabling us to assign them to established Nucleocytoviricota lineages. The placement of our MAGs within multiple Nucleocytoviricota Orders, including deeply divergent lineages, indicates that our newly identified MAGs are related to known viruses and extend the diversity of established Orders into freshwaters (Fig. 1). However, the recovery of distinct subclades comprised primarily of our MAGs, especially within the Imitervirales, suggests the potential for novel lower-rank lineages (i.e. within Orders) that may be affiliated uniquely with freshwaters.

The Orders *Imitervirales* and *Pimascovirales* accounted for most of the recovered MAGs (Fig. 1) and were dominant in most lakes (68 and 17 lakes, respectively). The most abundant Nucleocytoviricota MAGs in the dataset were two closely related *Imitervirales*, each recruiting ca. 6% of reads that mapped to Nucleocytoviricota across the entire dataset (Supplementary Data 5). Importantly, Nucleocytoviricota read recruitment varied widely among lakes and was not correlated with sequencing depth (ρ = 0.03, p-value = 0.781), suggesting that differences in Nucleocytoviricota abundance were independent of sequencing effort.

**Fig. 2:**
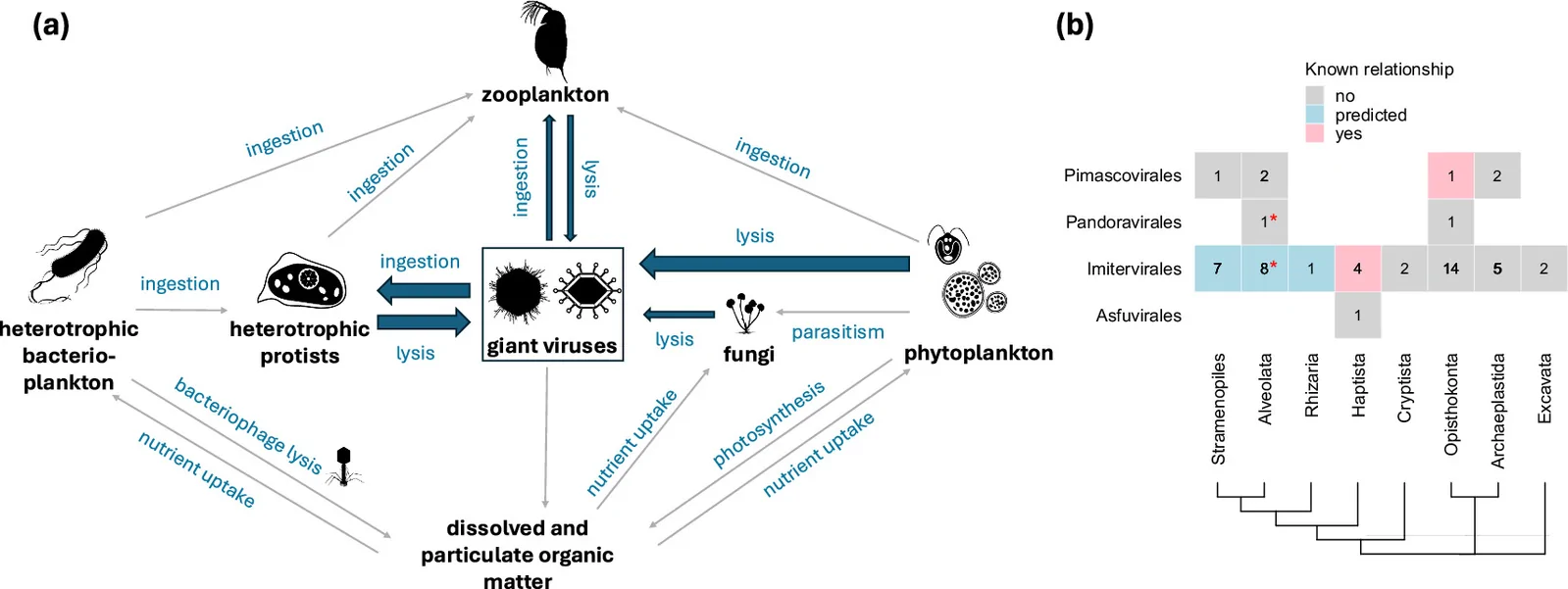
Nucleocytoviricota are highly connected to lake food webs. **a** Arrows indicate established direction of energy flow from source to sink with blue text denoting primary mechanism for the connection^99–101^. Dark blue arrows indicate connections established in this study, with their width proportional to the number of observed connections. Mixotrophic protists were included in heterotrophic protists and phytoplankton categories for simplicity. **b** Connections between Nucleocytoviricota Orders (vertical axis) and Eukaryote clades (horizontal axis) found in this study. Numbers represent the number of positive connections (FlashWeave edge weight measure >0.25), and the red star signifies strong evidence of horizontal gene transfer.

To predict Nucleocytoviricota-host interactions and their connections to freshwater food webs, we performed a co-occurrence analysis of our 87 lake metagenomes. This analysis identified 54 putative virus-host connections (Fig. 2, Supplementary Data 6) – the largest such database in lakes, to date. Most (85%) of the discovered connections were supported by at least some evidence of HGT, with only 43% of the connections having been previously reported or predicted in the literature (Supplementary Data 6). Most hosts were phytoplankton (32%), followed by heterotrophic (26%) and mixotrophic protists (11%) and zooplankton (11%) (Fig. 2). For the first time, two *Mesomimiviridae* MAGs from the genus *g336* were reported to co-occur non-randomly with a Ulvophyceae, while another *g336* MAG co-occurred with Chlorophyceae (Supplementary Data 6). To support the Mesomimiviridae-Ulvophyceae association, we identified functionally relevant host genes in viral MAGs (Supplementary Data 7), including those involved in polyamine biosynthesis, suggesting modulation of host growth and viral replication; dTDP-L-rhamnose biosynthesis, potentially influencing cell surface structure; and deoxyribonucleotide and pyrimidine biosynthesis pathways that would support viral genome replication. Additionally, we identified genes associated with glycosaminoglycan biosynthesis that may contribute to host cell surface modification, facilitating viral attachment or spread. We also identified novel protist-Nucleocytoviricota interactions, including between an Alveolate and *Pandoravirales* supported by strong HGT evidence, and five new connections with zooplankton in the families Calanoida, Cyclopoida and Diplostraca, Maxillopoda and Nucleocytoviricota MAGs from *Mesomimiviridae* and *Allomimiviridae* (Supplementary Data 6). Finally, 13% putative connections were between fungi and Nucleocytoviricota (Fig. 2), five of which included fungi that parasitise algae in the Aphelidea, Chytridiomycota, and LKM11 clades (Supplementary Data 6). Overall, the proportion of identified Nucleocytoviricota hosts per lake that were primarily autotrophs decreased with increasing latitude, likely reflecting latitudinal shifts to heterotrophy (ρ = −0.32, p-value = 0.005). To verify the robustness of our network analysis and account for potential undersampling inherent to the metagenomic approach, we repeated the network analysis using PolB (Supplementary Data 8) together with eukaryotic 18S rRNA genes. Networks were not significantly different in the number of hosts identified (Fisher’s simulated p-value = 0.114 based on 2000 replicates) (Supplementary Data 6; Supplementary Fig. 2), supporting the robustness of our conclusions.

### Biogeographic distribution of Nucleocytoviricota and their predicted functions

**Fig. 3:**
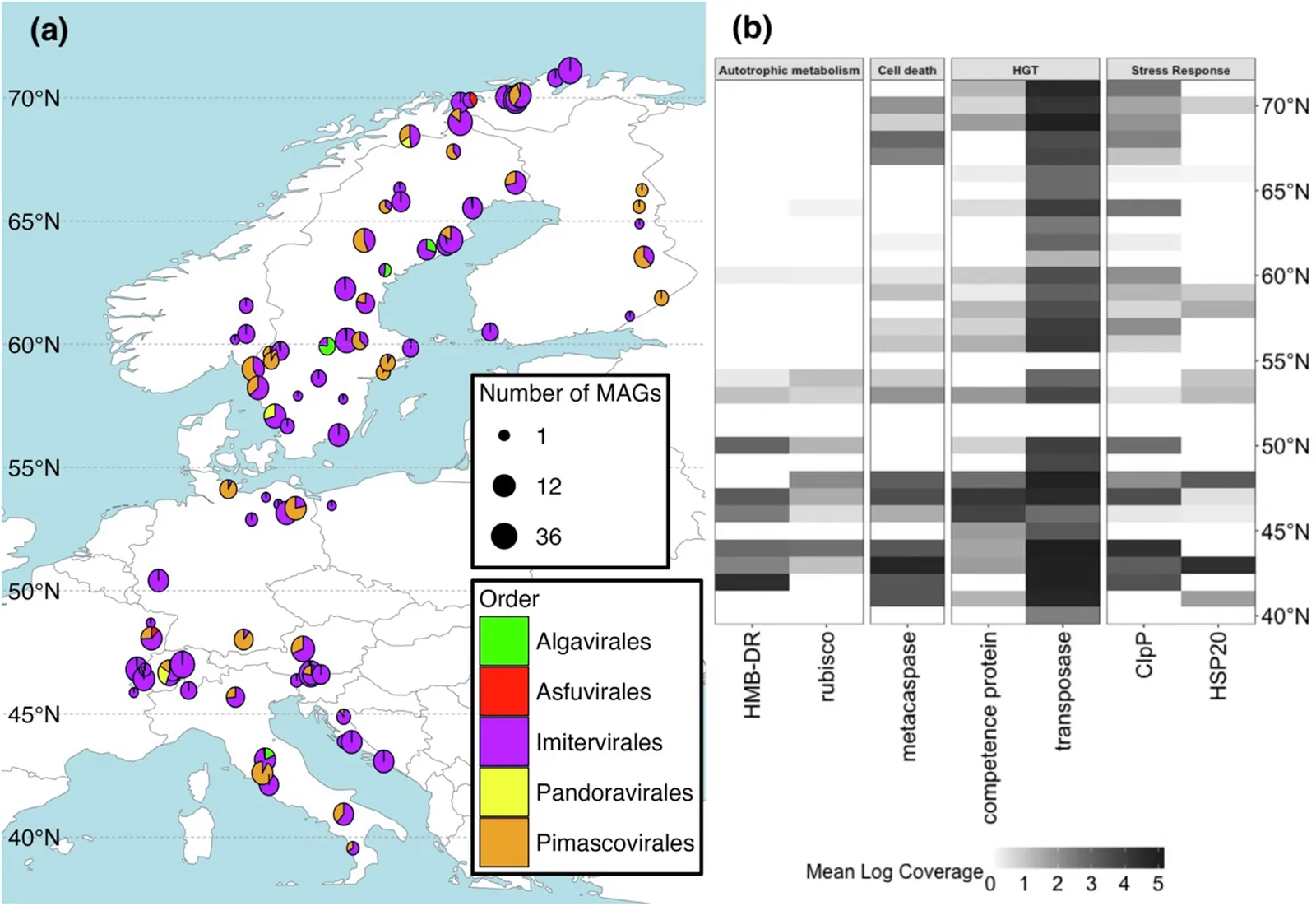
Taxonomic and functional distribution of nucleocytoplasmic large DNA viruses (Nucleocytoviricota) across European lakes. **a** Number of metagenome-assembled genomes (MAGs) for Nucleocytoviricota and the relative abundance of assigned taxonomic Orders recovered in each of 87 lakes. **b** Latitudinal distribution of Nucleocytoviricota functional genes. Latitude was rounded to the nearest integer and functional gene coverage (number of times each nucleotide was covered by metagenomic reads) was averaged in each 5° latitudinal bins. Grey facet strips are gene functional groups. HMB-DR: 4-hydroxy-3-methylbut-2-en-1-yl diphosphate reductase; rubisco: ribulose-bisphosphate carboxylase large chain; metacaspase: metacaspase-1; competence protein: competence protein CoiA; transposase: putative transposase; ClpP: ATP-dependent Clp protease, protease subunit; HSP20: HSP20 family protein.

We found a greater relative abundance but not richness of Nucleocytoviricota at southern latitudes, partly contrasting expectations from the latitudinal biotic interaction hypothesis^25^. The relative abundance of Nucleocytoviricota reads in each lake, expressed as a percentage of all metagenomic reads, averaged 0.24 ± 0.05% (mean ± SE), and nearly tripled in lakes south of the North Sea ( < 55°N) compared to northern lakes on the Scandinavian peninsula ( > 55°N) (Wilcoxon test, W = 652, p-value = 0.031; Fig. 3a). However, there was little evidence of a continuous decline in relative abundances with latitude (ρ = −0.19, p-value = 0.076), potentially reflecting broader differences in biogeographic history between southern and central ( < 55°N) versus northern Europe ( > 55°N). In southern and central Europe, Nucleocytoviricota were more abundant in lakes located at higher elevations (ρ = 0.38, p-value = 0.028, *n* = 34), whereas this trend was absent in northern Europe (ρ = −0.08, p-value = 0.556, *n* = 53). The relative abundance of Nucleocytoviricota also correlated with the log-ratio of PolB to 18S rRNA gene reads in each sample as an alternate metric of giant virus enrichment (ρ = 0.38, p-value = 0.002). By contrast, there was a similar richness of Nucleocytoviricota MAGs across our latitudinal gradient, ranging from 1 to 36 MAGs per lake (ρ = 0.027, p-value = 0.801) and between southern and central ( < 55°N) and northern ( > 55°N) lakes (W = 850, p-value = 0.660). There was also no association between Nucleocytoviricota richness and elevation in either southern and central Europe (ρ = 0.32, p-value = 0.064) or northern Europe (ρ = −0.08, p-value = 0.572). Although Nucleocytoviricota occurred across our large spatial gradient, they varied locally in abundance, as their composition varied with latitude at the level of MAGs (permutational multivariate analysis of variance: R^2^ = 0.02; p-value = 0.021) but not at the Order-level (R^2^ = 0.01; p = 0.510). Lakes that were more compositionally distinct were more distant from each other geographically (Mantel correlation: ρ = 0.11, p-value = 0.001) and were marginally more dissimilar in environmental characteristics (ρ = 0.08, p-value = 0.051; Supplementary Fig. 1). These results collectively demonstrate clear biogeographic structure in the abundance but not richness of Nucleocytoviricota across European lakes that may influence their functions and responses to future environmental change.

At a genomic level, we found that the functional gene repertoire of NCLDVs shifted across the latitudinal gradient, consistent with the prediction that lower latitudes were more favorable for replication and host infections. In southern and central European lakes, MAGs showed more genes with functions connected to autotrophic energy acquisition (Fig. 3b). The depth of coverage of the ribulose-bisphosphate carboxylase gene encoding the enzyme involved in the Calvin cycle decreased across the extent of our latitudinal gradient by 9.0% ± 1.0% (linear model: z = −2.45, p-value = 0.014) (Fig. 3b). At lower latitudes, we also found greater protection against stress, such as from a more frequent presence of genes encoding heat shock protein 20 (z = −3.09, p-value = 0.002) and the ATP-dependent caseinolytic protease proteolytic subunit (z = −2.40, p-value = 0.016), which degrades misfolded or damaged proteins^26^. We also found evidence of potentially enhanced efficiency of viral lysis at lower latitudes^27,28^, as inferred from a greater coverage of the metacaspase-1 gene (z = −2.93, p-value = 0.003; Fig. 3b).

**Fig. 4:**
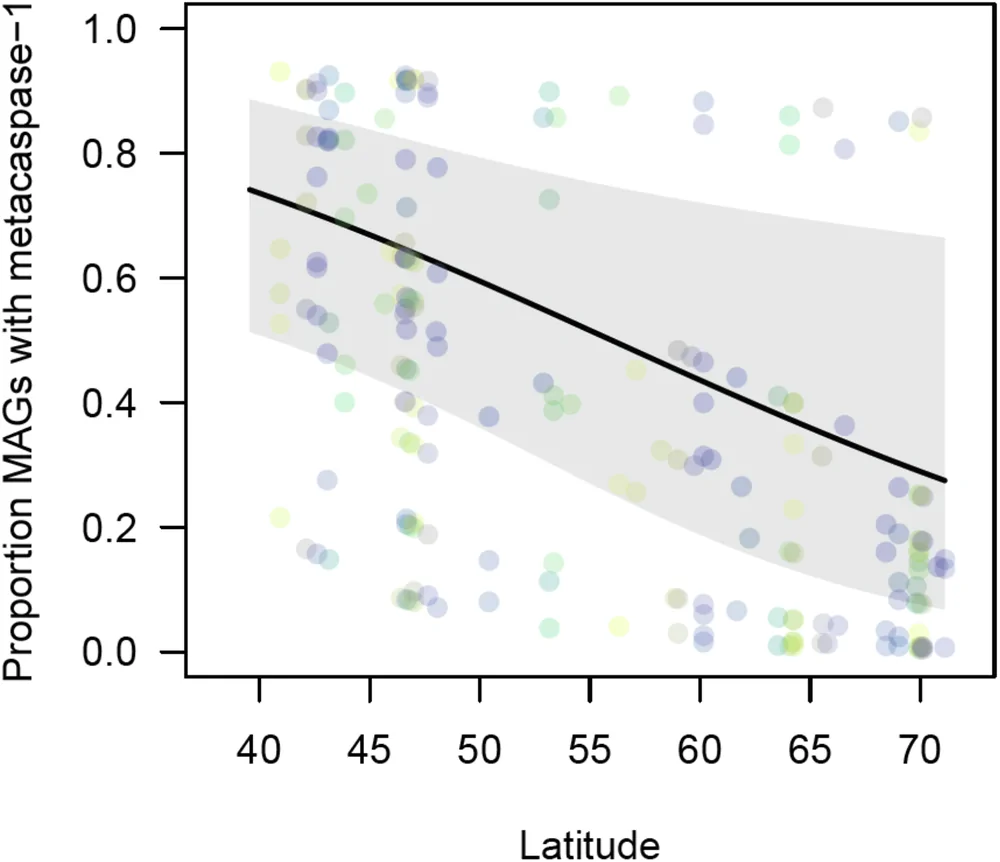
Intra-taxonomic variation drives latitudinal patterns in the presence of the metacaspase-1 gene. Coloured points represent the proportion of MAGs within each phylogroup estimated to contain the metacaspase-1 gene, with different colours representing individual phylogroups. The line indicates the model-estimated mean trend across latitude and phylogroups ± 95% confidence interval. Intra-taxonomic variation in functional gene distribution occurs where the proportion of MAGs containing the functional gene changes within a phylogroup with latitude, whereas inter-taxonomic variation was defined by a latitudinal gradient in the presence of phylogroups containing a functional gene.

To determine if the spatial differences in functional genes coverage arose because Nucleocytoviricota adapted to their environment or simply varied in abundance, we analysed the latitudinal distribution of functional genes within species by clustering MAGs into monophyletic groups (Fig. 1). We focused on phylogroups that contained >1 MAG and functional genes whose coverage varied with latitude, i.e. those with statistically significant trends (Fig. 3b). For six out of the seven modelled functional genes, we found that the observed latitudinal trend in their coverage could be attributed to variation in phylogroup abundance rather than intra-taxonomic differences (Supplementary Data 9). Across all the analysed phylogroups, there was no latitudinal variation in the proportion of MAGs containing a particular function (Supplementary Data 9). The only exception was the metacaspase-1 gene. The proportion of MAGs containing metacaspase-1 within individual phylogroups decreased by 35.5% ± 25.6% from southern to northern latitudes (z = −2.32, p-value = 0.021; Fig. 4). This finding suggests that more favourable environmental conditions, such as warmer long-term average temperatures and higher nutrient availability, may select for phylogroups containing genes that enhance viral replication and host cell death, e.g. metacaspase-1^28,29^. In our study lakes, total nitrogen concentrations declined with increasing latitude (ρ = −0.62, p-value < 0.001), consistent with previously reported global patterns in lake nitrogen and phosphorus concentrations^30,31^.

### Ecosystem-level effects of freshwater Nucleocytoviricota

**Fig. 5:**
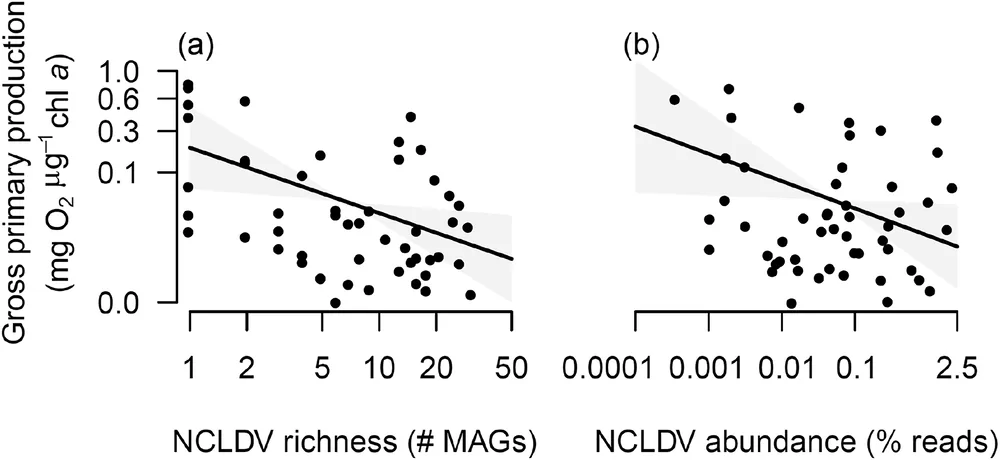
Primary production of European lakes decreases with nucleocytoplasmic large DNA viruses (Nucleocytoviricota). We measured gross primary production using bottle incubations and separately modelled how it varied with the **a** richness and **b** relative abundance of Nucleocytoviricota. Each model included the effect of Nucleocytoviricota alongside bacterial protein production, water temperature, maximum lake depth, specific ultraviolet absorbance, proportion of cropland in the catchment, and either latitude or whether sites were in southern and central (< 55°N) or northern Europe ( > 55°N). Results were plotted from the best supported model based on AICc (Supplementary Data 10). Points are lake-level observations (n=51), lines are the estimated mean trend, and shading is 95% confidence interval.

We detected ecosystem-level consequences from the biogeographic variation in the taxonomic and functional composition of Nucleocytoviricota that may intensify with global change. We assessed energy flow through lake food webs by measuring gross primary productivity (GPP) using bottle incubations. We found that GPP could be predicted (model R^2^ = 0.41) from Nucleocytoviricota richness (t_43_ = −2.64, p-value = 0.011), alongside other important environmental covariates, i.e., water temperature at the time of sampling, bacterial productivity, and surrounding land use (Supplementary Data 10). As Nucleocytoviricota richness increased over the observed inter-quartile range of values, GPP decreased by 8.0-times from an estimated mean (95% confidence interval) of 0.08 (0.04-0.14) to 0.01 ( < 0.01-0.03) mg O_2_ µg^−1^ chlorophyll *a* (Fig. 5a). The effect of Nucleocytoviricota richness on GPP was stronger than the effect of bacterial productivity or land use (namely cropland abundance) but not water temperature (Supplementary Data 10). We fitted the same model using the relative abundance of Nucleocytoviricota instead of richness. This model also revealed a negative effect of Nucleocytoviricota abundance on GPP (t_43_ = −2.23, p-value = 0.031), but this model was less supported than the one with richness (ΔAICc=2.1; Supplementary Data 10).

Nucleocytoviricota richness in each lake, and thus its effect on GPP, varied along gradients representative of future global change (Supplementary Fig. 3). We predicted per-lake Nucleocytoviricota richness from lake characteristics and environmental variables known to affect species richness (model R^2^ = 0.43, see Methods). We found that the per-lake Nucleocytoviricota richness increased with increasing values for the per-lake number of eukaryotic families (z = 3.75, p-value < 0.001), lake depth (z = 3.51, p-value < 0.001), and specific UV absorbance (SUVA), a measure of dissolved organic matter aromaticity (z = 2.95, p-value = 0.003). Richness also decreased with water temperature at the time of sampling (z = −2.45, p-value = 0.014). We obtained similar results when predicting relative abundance (Supplementary Data 10). Because the effect sizes of temperature and SUVA were similar (Supplementary Data 10), the decrease in per-lake Nucleocytoviricota richness over the observed range of water temperature was offset by the increase in Nucleocytoviricota richness over the observed range of SUVA. For example, our model implies that a 1.5 °C increase in water temperature alone can reduce NCLDV richness such that GPP could increase by 14%, on average, from a mean of 0.035 (95% confidence interval: 0.014-0.077) to 0.040 (0.016−0.087) mg O₂ µg chlorophyll *a*⁻¹ day⁻¹.

## Discussion

We uncovered numerous novel associations between Nucleocytoviricota and eukaryotic hosts spanning all trophic levels, reshaping our understanding of their ecological roles. Although the absolute richness of MAGs was relatively low, it was comparable to other studies on a per-sample basis^3,5^, and any underestimation would have been systematic across samples, thereby preserving relative differences in community interactions and functional genes. Notably, we found a broader diversity of Nucleocytoviricota lineages, particularly in the *Imitervirales* and *Pimascovirales*, may infect more freshwater phytoplankton than previously recognised. This result may reflect the widespread distribution of freshwater hosts susceptible to *Pimascovirales* infection, including known hosts such as freshwater amoebae and invertebrates^32–34^, as well as newly inferred hosts such as Alveolates and Chlorophytes. A particularly surprising finding was that 22 of 32 *Pimascovirales* MAGs belonged to the ecologically uncharacterised family *PM_01*, while the remainder could not be assigned to any known family. *PM_01* has only been reported in a few marine and freshwater metagenomic studies, and the range of their hosts is still unknown^7,24,35,36^. In our dataset, *Pimascovirales* MAGs were associated with a diversity of eukaryotic groups, including algae (Zygnematophyceae, Chlorophyceae, Fragilariaceae), protists (Choreotrichia, Mesodiniidae), and zooplankton (Diplostraca). These diverse connections suggest that Pimascovirales may occupy a broader ecological niche than previously recognised. Finally, we found new connections between Nucleocytoviricota and early-diverging fungal groups, such as Cryptomycota (LKM11), Chytridiomycota, and Aphelidea. Only eight Nucleocytoviricota infecting three fungal phyla have been described to date^37^, and Nucleocytoviricota co-occurrence studies in marine environments only detected one potential case of a fungal host^38^. An important caveat of our study is that co-occurrence does not constitute direct evidence of virus–host interactions, as taxa may simply share the same habitat or ecological niche^39^. Approximately half of the putative interactions we detected were supported by secondary evidence, such as signatures of horizontal gene transfer or previously described culture-based or predicted host–virus associations^6^ (Supplementary Data 6). Given that only a small fraction of Nucleocytoviricota genomes have been linked to confirmed hosts, most of the associations identified in our study require further validation, such as with PCR. For example, the early diverging fungal groups are primarily known as algal parasites^40^. These associations could therefore reflect indirect, tripartite interactions involving Nucleocytoviricota, fungal parasites, and their shared algal hosts.

Our results suggest that differences in environmental characteristics led to spatial variation in Nucleocytoviricota, potentially by changing host status and availability. First, high attenuation of UV radiation can promote the formation of reactive oxygen species^41^, which can increase the susceptibility of algae to Nucleocytoviricota infections^42^. Freshwater protists, which comprised one-third of the hosts in our dataset, are also sensitive to UV damage^43,44^. Any resulting damage could thus render protists in the epilimnion more susceptible to Nucleocytoviricota infections, as observed in algae^42^, and increase the richness and abundance of Nucleocytoviricota in our surface samples. Second, lakes with higher SUVA, indicative of more complex, aromatic carbon sources, generally support higher abundances of aquatic fungi that preferentially degrade these substrates and could offer more hosts for Nucleocytoviricota^45,46^. In support of this explanation, SUVA in northern Europe was nearly double that in southern and central Europe (t_56_ = 5.26, p-value < 0.001), and these lakes had the most connections between Nucleocytoviricota and fungi (Supplementary Data 10). The increase in SUVA with elevation in southern and central Europe (ρ = 0.36, p-value = 0.049), but not in northern Europe (ρ=0.03, p-value=0.827), could similarly explain the differences in how Nucleocytoviricota varied with elevation between regions. Third, lake depth was the strongest predictor of Nucleocytoviricota richness and abundance, potentially because deeper waters are more physically stable, thereby enabling Nucleocytoviricota to persist and accumulate on their hosts^47^. In support of this explanation, others have found higher compositional turnover over time for Nucleocytoviricota in epilimnetic waters than in the more stable hypolimnion^8^. Finally, Nucleocytoviricota richness and abundance declined with increasing water temperature, in contrast to our predictions. This result is consistent with the lack of consensus in marine studies on how Nucleocytoviricota diversity varies with temperature or latitude^3,35,48^, though many unique Nucleocytoviricota phylotypes are associated with cold waters^3^. During the period when we sampled our study lakes, high temperatures could have reduced algal diversity^49,50^, and thus reduced potential Nucleocytoviricota hosts. Taken together, our study suggests that deep, cold lakes with high aromatic carbon content and diverse eukaryotic communities are likely biodiversity hotspots for Nucleocytoviricota.

We also found that the distribution of Nucleocytoviricota functional genes reflected shifts in host composition (Supplementary Data 11). Genes that can enhance autotrophic energy acquisition and stress mitigation, as well as genes potentially involved in the transformation of host genomes and cell death, were all more abundant in the southern and central lakes, which coincided with a greater proportion of autotrophic hosts, such as algae. Thus, Nucleocytoviricota may shift towards reprogramming host metabolic pathways in lakes dominated by autotrophic activity to enhance their own energy acquisition and replication efficiency during infection. Previous studies have shown that Nucleocytoviricota gene expression linked to photosynthesis and carbon metabolism is upregulated in autotrophic hosts^19^. Concurrently, these Nucleocytoviricota and their hosts may be more exposed to thermal stress at central and southern latitudes, which could explain the increased coverage of genes involved in stress responses^20^. The temperature tolerance of viruses is typically broader than that of their hosts^51^, making it advantageous for Nucleocytoviricota to reprogram their hosts to be more heat tolerant. Finally, the warmer and more nutrient-rich environments in southern and central Europe may have promoted rapid lytic replication and the prevalence of genes like metacaspase-1 by enhancing viral replication, even within the same phylogroup. In contrast, in cooler and more nutrient-limited environments that can both limit host availability and metabolism, as well as slow virion assembly, viruses might opt for a lysogenic strategy to maximise persistence and replication^52^. These associations between latitude and nutrients in lakes contrast with marine systems, where enhanced vertical mixing sustains high nutrient concentrations at high latitudes. In lakes, nutrient supply is primarily controlled by terrestrial catchments and climate, which slow nutrient cycling at higher latitudes^53^. More generally, our results underscore how local environmental conditions influence viral ecology, particularly energy acquisition, stress protection, and infection efficiency, and may thereby shape wider food web dynamics.

Much of our understanding about the host associations, biogeography, and functional gene composition of Nucleocytoviricota communities is derived from the oceans, where environmental filters and dispersal processes shape distinct patterns from freshwaters. For example, lakes receive proportionally greater inputs of terrestrial organic matter than marine environments, especially the open ocean^54^, resulting in chemically different substrates for eukaryotic consumers. Consequently, lakes may offer different ecological niches for hosts that influence Nucleocytoviricota distributions, and we detected frequent associations between Nucleocytoviricota and both zooplankton and fungi that are less apparent in the oceans^54^. Lakes also differ from marine environments, especially the open ocean, because weather-driven mixing can rapidly redistribute organisms across depths and restructure encounter rates between viruses and their hosts, in contrast to the more persistent circulation patterns of oceans^1^. This mechanism is consistent with our observation that Nucleocytoviricota richness and abundance were better explained by lake-specific characteristics, such as depth and organic matter quality, rather than by latitude alone. In marine systems, Nucleocytoviricota composition is strongly structured by basin and biome because it tracks major water masses and distributions of primarily photosynthetic planktonic eukaryotes, resulting in large-scale biogeographic patterns^5^. By contrast, the discrete catchment boundaries of our study lakes may impose stronger dispersal limitation and compositional differences over small spatial scales, as reflected by the positive correlation between Nucleocytoviricota compositional dissimilarity and geographic distance. Finally, while marine Nucleocytoviricota commonly encode AMGs involved in metabolism and stress responses that align with broad oceanographic gradients^55^, our results suggest that their distribution in lakes primarily reflects local environmental conditions, such as nutrient availability, light availability, and thermal structure.

Our results suggest that, by overlooking Nucleocytoviricota, ecosystem models risk inaccurately predicting biogeochemical cycling and food web dynamics in freshwaters. Using our statistical models, we found that a 30% increase in SUVA, as observed in other European catchments over the past three decades^56^, was large enough to reduce GPP by a mean of 24% (95% confidence interval: 6-39%), given the corresponding increase in Nucleocytoviricota richness for a lake with mean values of all other variables in our models. When accounting for future climate warming, the negative effect of water temperature on Nucleocytoviricota richness was effectively offset by its positive effect on GPP (Supplementary Data 10). A 1.5 °C increase in water temperature, as expected from future climate warming^57^, therefore reduced GPP by 20% (5-33%) when forecast alongside the 30% increase in SUVA. These coarse extrapolations demonstrate that ecosystem models that overlook the effects of Nucleocytoviricota cannot predict a large amount of variability in lake productivity. As widespread darkening of waters is predicted under a warmer, wetter climate^58^, our results suggest that the impact of Nucleocytoviricota on future whole lake productivity will depend on the magnitude of warming that can offset the effects of SUVA. More broadly, these results help to explain why the viral responses to warming temperatures might vary rather than be consistent across environments^59,60^. Our study now underscores the importance of integrating viral ecology into broader ecological and biogeochemical frameworks to predict the future of Earth’s freshwaters.

## Methods

### Field sampling

Surface water grab samples (0.5 m depth) were collected from April to September 2019 from 87 lakes located between 39°N and 71°N. A single sample was collected from above the deepest point of each lake (1–322 m), and 1 L of water was immediately filtered through a Sterivex filter (polyethersulfone membrane, 0.22 um pore size, Millipore, USA). If the filter became clogged (ca. 5% of samples), we continued filtering with a new Sterivex until a total 1 L of water was sampled and we sequenced the multiple filters and pooled reads downstream (see next section). Filters were immediately frozen (−20 °C) and stored at −80 °C within 14 days. We collected the filtrate inside pre-combusted (450 °C, 4 h) borosilicate vials for estimating organic matter concentration and optical properties. Briefly, we measured absorbance from 190 nm to 800 nm (1 nm increment) using a Flame UV-Vis spectrometer (OceanOptics, USA). We measured dissolved organic carbon and total dissolved nitrogen concentration using a TOC-V analyzer (Shimadzu, Japan). Then, we calculated the specific UV absorbance (SUVA) by dividing the absorbance at 254 nm by the dissolved organic carbon concentration. We immersed a multiprobe (HI-99171, Hanna Instruments) inside each lake to measure pH. We estimated bacterial protein production (BPP) using labelled leucine^61^ as described previously^62^. Briefly, we incubated 1.5 mL of lake water in-situ with 17 nM of [^3^H]-leucine for 1 hour and either 300 μL of distilled water (“live”) or 300 μL of trichloroacetic acid (“dead”). We retrieved counts of the number of radioactive decay events per minute on site using a Triathler liquid scintillation counter (Hidex Oy, Finland) and calculated BPP as the difference in counts per minute between live and dead samples. Finally, we inferred phytoplankton biomass from total chlorophyll *a* concentration, which was measured in situ using fluorescence excitation at 370, 470, 525, 570, 590, and 610 nm with a fluorometer (bbeMoldaenke, Kiel-Kronshagen, Germany).

In the lab, we measured gross primary production (GPP)^63^ by incubating 20 mL of unfiltered lake water in duplicate glass bottles with no headspace for 3 hours at room temperature under a cold 5000 K lamp that emitted ca. 34,000 lux. Bottles were sealed gas-tight with rubber lids and a crimp top. We recorded dissolved oxygen concentrations at the start and end of the incubation in each bottle with an optical sensor (OXY-1 ST, PreSens, Germany). GPP rates were calculated from the difference between dissolved oxygen concentrations at the start and end of the incubation, averaged across the duplicate bottles for each lake. GPP rates were normalised by phytoplankton biomass inferred from chlorophyll *a* concentration in each lake after Regaudie-de-Gioux and Duarte^64^.

### Metagenomic sequencing

DNA was extracted according to Griffiths et al.^65^ with modifications in Sheridan et al.^62^. Briefly, filters underwent bead-beating in cetyltrimethyl-ammonium bromide buffer with anionic surfactants and phenol-chloroform-isoamylalcohol mixture. After centrifugation and phase separation, DNA was precipitated in 30% polyethylene glycol 6000, washed in ethanol and dissolved in nuclease-free water.

Shotgun metagenomic sequencing libraries were prepared using the NEBNext Ultra II FS kit (New England Biolabs, USA) following the manufacturer’s instructions. Briefly, DNA was digested for 15 min to obtain end-polished and A-tailed fragments of ~350 bp. Illumina adaptors were attached by incubating the fragments at 37 °C for 15 min in the presence of a ligation enhancer. Samples were cleaned using AMPure XP beads (Beckman Coulter, USA) and Illumina-specific indexes (New England Biolabs, USA) attached. We then purified the product using AMPure XP beads, quantified DNA concentration using a Qubit fluorometer (Thermo Fisher, USA), and pooled libraries at equimolar concentrations. Libraries were sequenced on a NovaSeq 6000 PE 250 bp (Illumina, USA) at Novogene UK, who performed demultiplexing and checked read quality (Q30 > 75%, Q20 = 100%).

### Nucleocytoviricota MAGs mining and characterisation

Metagenomes were assembled by lake individually using metaSpades v3.15.3 with the default parameters, including the initial step for read quality control^66^. Binning of assembled contigs was performed for each assembly individually (contigs > 2000 bp) with MetaBAT 2 v.1.7 using default parameters^67^. Assembly and binning steps were performed in KBase^68^, resulting in 58,938 bins.

GVClass v1.1.1^69^ was used to identify Nucleocytoviricota MAGs in metagenomic bins by detecting and scoring hallmark viral genes, applying phylogenomic thresholds, and classifying MAGs based on curated viral marker gene profiles. Specifically, initial gene calling (gene prediction) of the MAGs was performed with prodigal -p meta v2.6.3^70^. To identify candidate Nucleocytoviricota MAGs, proteins from each MAG were analyzed with hmmsearch (http://hmmer.org, version 3.4) against seven Hidden Markov Models (HMMs) built from seven Nucleocytoviricota orthologous groups (GVOGs): SFII, RNAPL, PolB, TFIIB, TopoII, A32, and VLTF3^6^. MAGs with more than one detected GVOG (E-value cut-off = 1e-5) were selected for further QC with GVClass v1.1.1^69^. This analysis also included revised gene calling in which the genetic code (out of 1, 4, 6, or 11: https://www.ncbi.nlm.nih.gov/Taxonomy/Utils/wprintgc.cgi) was selected that produced the highest coding density (percentage of base pairs predicted as part of a coding sequence in the Nucleocytoviricota MAG). Nucleocytoviricota MAGs with GC content outside the published range of 21–64%^71^ were individually checked by the default contig clustering and visualisation workflow of anvi’o v.7.1^72^. 15 Nucleocytoviricota MAGs were refined by being split into separate Nucleocytoviricota MAGs based on contig clustering and removal of noisy contigs, resulting in 57 additional Nucleocytoviricota MAGs. After refinement, Nucleocytoviricota MAGs were again run through the gvclass pipeline for taxonomic annotation based on matching detected GVOGs against a reference taxonomy of Nucleocytoviricota^6^.

The final set of 208 MAGs identified in this study was de-replicated and clustered by average nucleotide identity (ANI) alongside 1384 Nucleocytoviricota reference genomes^24^ with skani v.0.3.1^73^. Species-level clusters were defined using a 95% ANI threshold, following recommendations for species-rank virus operational taxonomic units^74^ and a 70% aligned-fraction threshold. ANI clustering yielded 205 query-only clusters (i.e., no reference genome representatives and thus putatively novel species), 1383 reference-only clusters, and one mixed query-reference cluster.

Phylogenetic placement of the 206 query-associated MAGs discovered in this study was undertaken against the 1383 reference representatives using the NSGTree v0.6.5 pipeline. Marker searches were performed against the same GVOG7 marker set^24^ used for gvclass screening. Per-marker protein alignments were generated with MAFFT and trimmed with trimAl using ‘-gt 0.1‘. Genomes were retained in the concatenated supermatrix if at least 3 of the 7 GVOG markers were present, corresponding to the ‘--percent_models 42‘ threshold used in the run. Of 1589 initial genomes, 70 were removed by the marker-retention filtering, leaving 1519 genomes in the final supermatrix alignment. A maximum-likelihood tree was inferred from the concatenated amino acid supermatrix containing 7487 sites with IQ-TREE v3.1.1^75^ using 1000 ultrafast bootstrap replicates and 1000 SH-aLRT replicates. We used the Q.pfam+R10 + F model in IQ-TREE that was selected for the Nucleocytoviricota tree with ModelFinder as described in ref. ^76^.

Functional gene annotation was performed in anvi’o v.7.1^72^ by identifying KEGG Orthologs^77^ using KOfam HMM profiles (anvi-run-kegg-kofams), and additionally by eggNOG-mapper v.2.1.9^78,79^. Coverage information for the functional genes was calculated using anvi-export-gene-coverage-and-detection.

### Nucleocytoviricota marker gene validation

To identify potential Nucleocytoviricota marker genes that may have been missed using a MAG-centric approach, we first predicted protein-coding genes from all metagenomic assemblies using GVClass v1.1.1^69^. This step enabled the recovery of unbinned viral genes from the entire metagenomic dataset. The resulting amino acid sequences were then screened for the DNA polymerase B (PolB) subunit, a hallmark of Nucleocytoviricota^24^, using HMMER v3.3.2 and a curated HMM profile specific to viral PolB domains^80^. To verify the origin of these candidate PolB genes and minimise potential contamination from non-Nucleocytoviricota sources, we created a gene tree using Nucleocytoviricota PolBs and 2791 PolBs from other groups (1331 eukaryotes, 40 bacteria, 1217 archaea, 54 phages, 149 Mirusviricota), using veryfasttree v4.0.4.1^81^. All PolB sequences with a nearest neighbour identified as Nucleocytoviricota were retained.

### Abundance estimations for Nucleocytoviricota MAGs and Eukaryote contigs

The ssuextract pipeline v.0.9.0 (https://github.com/NeLLi-team/ssuextract) was used to identify, extract, and taxonomically annotate 18S rRNA genes of assembled contigs. Annotation was based on the best blastn hits in the SILVA release 138 and pr2 18S rRNA v. 5.1.2 databases^82,83^. To assess the geographic distribution of Nucleocytoviricota MAGs and eukaryotic 18S contigs across the study lakes, contigs were used as references for mapping of metagenomic reads. Mapping was performed using BBMap v.35.85 (http://sourceforge.net/projects/bbmap/) with the following parameters: minid=0.95, minaveragequality=30, slow=t, and ambiguous=random. CoverM v.0.6.1 (https://github.com/wwood/CoverM) in genome mode and samtools v.1.16^84^ were used to extract coverage metrics (depth of coverage, breadth of coverage, number of reads, relative abundance defined as proportion of mapped reads relative to total number of metagenomic reads). Eukaryotic contigs with >90% breadth of coverage and Nucleocytoviricota MAGs with >10% breadth of coverage were considered to be present in the corresponding sample^85^. The difference in this threshold arises from the two-order-of-magnitude difference in Nucleocytoviricota MAG size (mean = 379,602 bp, SD = 194,392 bp) compared with contigs that encoded the 18S rRNA gene (mean = 1344 bp, SD = 355 bp). An abundance of eukaryotic contigs was aggregated at the family level. Host trophic status was assigned based on the closest phylogenetic relative in a reference database^83^ containing curated ecological and functional annotations of eukaryotic 18S rRNA sequences. When classification was uncertain, taxa were conservatively assigned as mixotrophic.

### Network analysis to infer virus-host associations

Putative virus-host interactions were inferred based on co-abundance analysis of Nucleocytoviricota MAGs and eukaryotic contigs using FlashWeave v.1.6 run in sensitive mode^86^. FlashWeave tests the conditional independence of pairwise combinations of taxa, and only associations with a conditional independence test of p < 0.01 were considered statistically significant. The output from FlashWeave consisted of a weighted adjacency matrix, where each element represented the strength of an association between a contig and a MAG. Negative and weak positive (weight <0.25) associations between Nucleocytoviricota and eukaryotes were discarded, as well as any Nucleocytoviricota-Nucleocytoviricota and eukaryote-eukaryote associations. The remaining Nucleocytoviricota-eukaryote associations were investigated for evidence of HGT by performing a protein similarity search of Nucleocytoviricota gene calls against the Comparative Set of EukProt database v3^87^ using BLAST + 2.15.0^88^. Alignments with amino acid identity > 50%, query coverage > 50%, and e-value < 1e-50 were considered as candidates for HGT^4,6^. Candidate HGT query sequences were further aligned against the NCBI database [accessed on 6 Feb 2024] using protein BLAST to exclude any higher similarity matches to viruses or prokaryotes based on total score. Only the highest-scoring matches were considered strong evidence for horizontal gene transfer, while all other hits were treated as weak evidence (Supplementary Data 6).

To assess patterns of host-giant virus co-occurrences further, we linked Nucleocytoviricota to eukaryotes using PolB and 18S rRNA sequences as markers for each group, respectively. For Nucleocytoviricota, we used GVClass to identify PolB sequences by utilising HMMER and hmmsearch (-E 1e-10)^89^ with HMMs generated by GVClass (Supplementary Data 12). We recovered 1498 PolB sequences and confirmed that 1460 PolB sequences were phylogenetically placed with Nucleocytoviricota genomes based on the phylogenetic nearest-neighbour identified with GVClass. Using similar methods from Meng et al. 2021^4^, we then created a PolB and 18S rRNA gene matrix to perform network inference. We first applied a centred log-ratio transformation on each matrix separately, after adding a pseudo count of one to all values. We then input the combined matrix to Flashweave v.0.19.2 to identify putative connections between Nucleocytoviricota and eukaryotes (sensitive=true, heterogeneous=false, alpha = 0.01, Supplementary Data 8)^86^. For PolBs with putative eukaryotic connections, we used the nearest-neighbour results from GVClass to classify sequences at the Family-level.

### Statistical analysis of Nucleocytoviricota taxonomic composition

We tested how the taxonomic composition of Nucleocytoviricota varied with environmental factors. First, we tested how the richness and summed relative abundance of Nucleocytoviricota in each lake varied with latitude using Spearman rank correlations. Second, we tested how community composition varied with environmental conditions using Mantel tests of distance matrices with Spearman’s correlation coefficient and 999 permutations. Between all pairwise combinations of lakes, we calculated the Morisita-Horn dissimilarity index for the relative abundance of Nucleocytoviricota MAGs. We correlated this matrix with the Euclidean dissimilarity in 10 environmental variables that were each normalised to a mean of zero and standard deviation of one. The environmental variables were pH, dissolved organic carbon and nitrogen concentrations, SUVA, water temperature at the time of sampling, lake area, and phytoplankton primary productivity, measured as described above. Additionally, we retrieved catchment area and the proportion of both forest and urban area per catchment at a 100 m resolution by clipping the CORINE land cover database^90^ to the area of each catchment retrieved from the Global Lake area, Climate, and Population dataset^91^. Only 65 lakes had all parameters measured, so the dissimilarities were calculated only for this subset. We repeated this analysis by replacing the environmental matrix with the geographic distances between lakes. Distances were calculated using the Haversine formula in the R package ‘geosphere’ v.1.5.14, which derives the shortest distance between two points on a sphere^92^.

We assessed the effect of latitude on Nucleocytoviricota community composition by fitting a permutational multivariate analysis of variance using the adonis2 function in the vegan R package v.2.6.6^93^ to the Bray-Curtis dissimilarities between lakes in abundance. Latitude was the only explanatory variable, and statistical significance was determined using 999 permutations.

### Statistical analysis of functional gene distributions

We tested if and how functional genes varied across the latitudinal gradient using generalised linear models. We focused our analysis on 214 of 746 functionally characterised genes involved in autotrophic metabolism, HGT, or stress responses, and whose coverage significantly varied with latitude (Supplementary Data 9). Coverage of each gene per lake was calculated from the average number of aligned metagenomics reads overlapping each position on the gene. We then subset this list to select only those genes (*n* = 8) present in at least 8 lakes. A separate hurdle-Gamma model was fitted to the coverage of each functional gene with >10% non-zero values per lake using the R package glmmTMB v.1.1.7^94,95^. This model estimated the probability of detecting each functional gene across all our samples by fitting a Bernoulli function to the binary outcome of whether coverage was zero or positive, with the probability of a non-zero outcome predicted from the log-transformed number of all Nucleocytoviricota reads in a lake. A zero-truncated gamma distribution was fitted to the positive coverage values with latitude as the sole predictor. For genes that varied with latitude, we then tested whether these patterns arose from intra- or inter-taxonomic changes, that is, whether the prevalence of a functional gene changed within individual Nucleocytoviricota phylotypes or as Nucleocytoviricota composition changed. We clustered Nucleocytoviricota MAGs into phylogroups based on manual tree inspection and identification of monophyletic groups (Fig. 1, Supplementary Data 2), as assigning true species to MAGs and viruses is challenging^74^. We then calculated the ratio of the number of MAGs containing each functional gene to the number of MAGs where that gene was absent for every phylogroup in each lake. Any variation in this ratio across latitude would suggest that intra-taxonomic variation (i.e., changes in the distribution of the focal gene among MAGs in a phylogroup) explains the geographic variation in gene coverage. By contrast, the absence of an association between the ratio and latitude would imply that changes in gene coverage arise from inter-taxonomic variation (i.e., changes in MAG composition across latitude). To assess these two hypotheses, we predicted the ratio of each gene by latitude using generalized linear models with a binomial distribution to the number of MAGs in each phylogroup, with and without the focal functional gene, in each lake.

### Statistical analysis of ecosystem-level effects

We tested if gross primary productivity (GPP) varied with Nucleocytoviricota and environmental factors, and how Nucleocytoviricota themselves varied with environmental conditions, using generalised linear models. We fitted linear models to quarter-root-transformed GPP values using water temperature, SUVA, maximum lake depth, BPP, and the proportion of cropland in surrounding catchments as predictors. We expected GPP would depend on light penetration captured by SUVA, nutrient competition with bacteria, and nutrient inputs from surrounding land use^96,97^. We then compared four models that included additional predictors of either the richness or relative abundance of Nucleocytoviricota in each lake and either the latitude of each lake or whether it was located north or south of 55°N that divided lakes in northern Europe from central and southern Europe. Models were compared using the small-sampled Akaike Information Criterion (AICc), whereby models with lower AICc by >2 were more strongly supported^98^. We then predicted the log-transformed relative abundance of Nucleocytoviricota with a linear model and Nucleocytoviricota richness with a generalised linear model with a lognormal-Poisson distribution to account for overdispersed count data. Each model included maximum depth, SUVA, water temperature, and the number of eukaryote families present in each lake inferred from the 18S rRNA gene data. In all models, predictors were normalised to a mean of zero and standard deviation of one so that their effects were directly comparable.

### Reporting summary

Further information on research design is available in the Nature Portfolio Reporting Summary linked to this article.

## Supplementary information

Supplementary Information

Description of Additional Supplementary Information

Supplementary Data

Reporting Summary

Transparent Peer Review file

## Data Availability

Sequencing data used in this study have been deposited to ENA under project accession number PRJEB40644. Reference genomes of giant viruses are available here: [https://faylward.github.io/GVDB/]. All ecological data are available in Supplementary Data 1.
