# Supplementary material for: The biogeography of giant viruses and interactions with Eukaryotes across European lakes
Source: Nat Commun. 2026 Sep 23;17:11056. doi: 10.1038/s41467-026-77765-w (PMC13691094; doi:10.1038/s41467-026-77765-w)
Supplement: Supplementary file 1 [file 41467_2026_77765_MOESM1_ESM.pdf]

Supplementary Figures

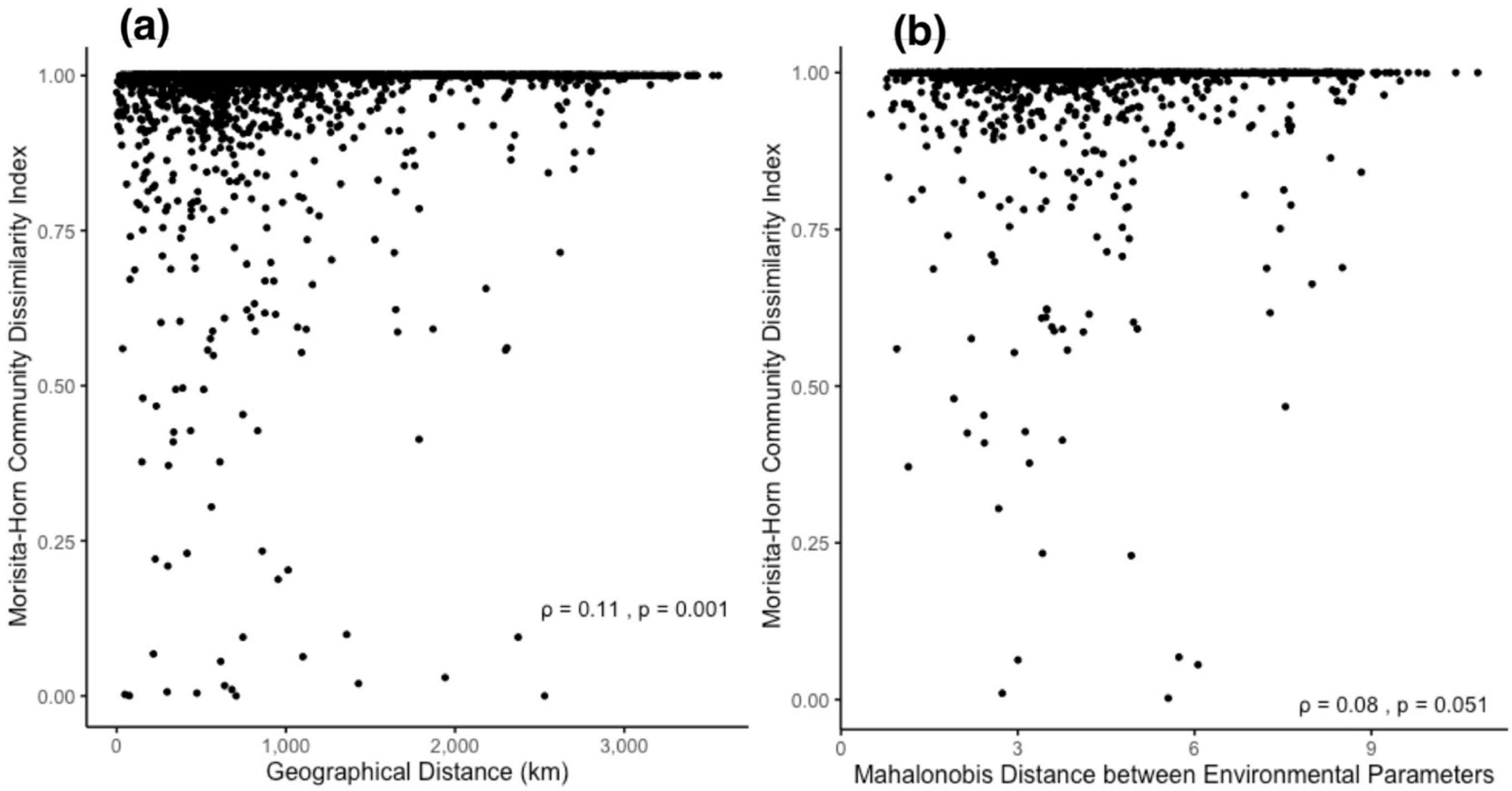

Supplementary Fig. 1. Nucleocytoviricota composition varies with geographic distance and environmental distance. Community dissimilarity (Morisita-Horn index) of Nucleocytoviricota at the MAG-level between all pair-wise combinations of lake samples (a) increased with geographic distance and (b) increased with environmental dissimilarity. Environmental dissimilarity was calculated based on the Mahalanobis distance between lakes for pH, dissolved organic carbon and nitrogen concentrations, SUVA, water temperature at the time of sampling, lake area, catchment area, proportion of forest and urban areas in the catchment, and primary productivity. For (a), n = 87, where n = 65 for (b) because environmental data were only available for a subset of lakes; ρ is the Mantel correlation coefficient and p is the p-value.

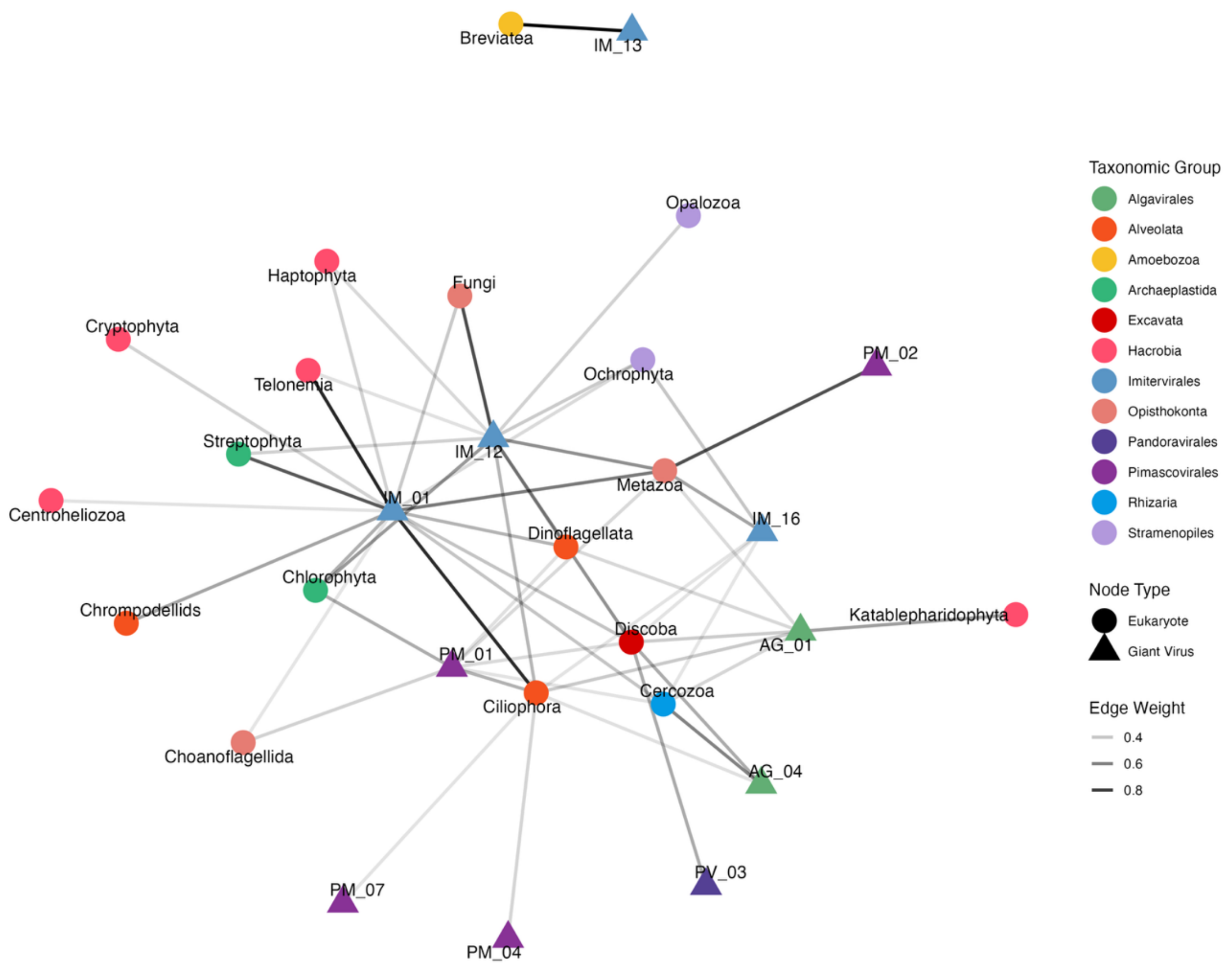

Supplementary Fig. 2. Co-occurrence network between Nucleocytoviricota DNA Polymerase B genes and Eukaryotic 18S rRNA genes. Nodes represent Nucleocytoviricota and eukaryotic contigs; edges indicate significant positive associations inferred with FlashWeave, with edge colour intensity proportional to association strength. The overall pattern of connections with major host groups is consistent across alternative network inference approaches, supporting the robustness of inferred virus–host relationships

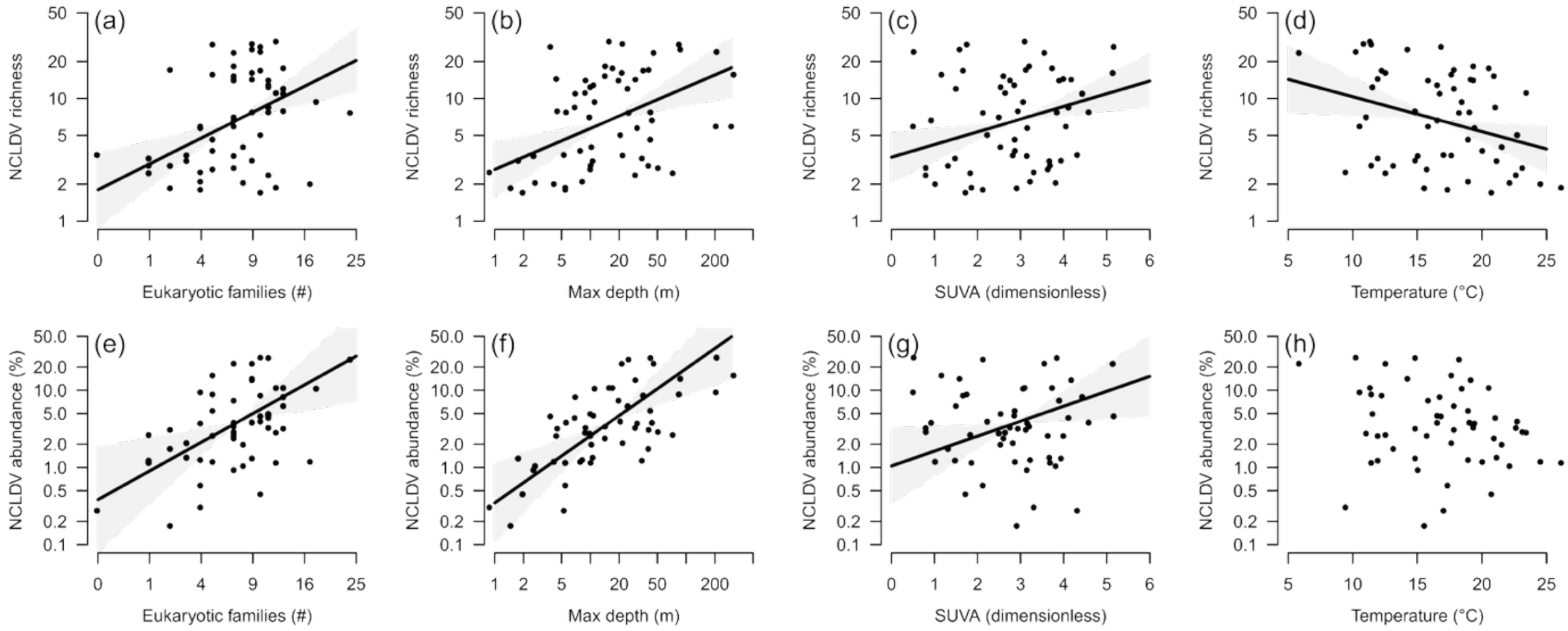

Supplementary Fig. 3. Nucleocytoviricota richness and abundance co-vary with eukaryote richness and environmental conditions. We separately modelled how the richness and relative abundance of Nucleocytoviricota varied in each lake varied with maximum depth, specific UV absorbance (SUVA) at 254nm, water temperature at the time of sampling, and the number of eukaryote families present in each lake inferred from the 18S rRNA gene data. Points are lake-level observations (n= 51), lines are estimated mean trend and shading is 95% confidence interval. Trends are only plotted where they were statistically significant (Supplementary Table 10).
