# Supplementary material for: The biogeography of giant viruses and interactions with Eukaryotes across European lakes
Source: Nat Commun. 2026 Sep 23;17:11056. doi: 10.1038/s41467-026-77765-w (PMC13691094; doi:10.1038/s41467-026-77765-w)
Supplement: Supplementary file 2 [file 41467_2026_77765_MOESM2_ESM.pdf]

Supplementary Data Legends

Title: Supplementary Data 1.
Description: Lake metadata. Parameters are explained in additional Metadata_parameters_1 spreadsheet.

Title: Supplementary Data 2.
Description: Nucleocytoviricota MAGs classified by gvclass [https://github.com/NeLLi-team/gvclass]. Parameters are explained in additional Metadata_parameters_2 spreadsheet.

Title: Supplementary Data 3.
Description: Average nucleotide identity (ANI) between Nucleocytoviricota MAGs.

Title: Supplementary Data 4.
Description: Nucleocytoviricota MAGs assessed by CheckV [https://github.com/cmarqu/checkv]. Parameters are explained in additional Metadata_parameters_4 spreadsheet. CheckV is currently the best method, to our knowledge, to assess MAG completeness and contamination despite being less reliable for Nucleocytoviricota. CheckV was designed and benchmarked for smaller DNA viruses and bacteriophages rather than the very large, complex, and often highly divergent genomes of Nucleocytoviricota, many of which are poorly represented in CheckV's reference database. Consequently, CheckV often underestimates Nucleocytoviricota genome completeness or incorrectly flags genuine viral sequences as contamination.

Title: Supplementary Data 5.
Description: Nucleocytoviricota community composition matrix. Columns are lake codenames matching those given in Title: Supplementary Data 1. Cells contain the percentage of Nucleocytoviricota reads per metagenome assembled genome (rows) per total number metagenomic reads generated per sample.

Title: Supplementary Data 6.
Description: Connections between Nucleocytoviricota MAGs and 18S rRNA eukaryotic contigs. Parameters are explained in additional Metadata_parameters_6 spreadsheet.

Title: Supplementary Data 7.
Description: Functional analysis of Nucleocytoviricota MAG contigs by EggNog [https://github.com/eggnogdb/eggnog-mapper]. Parameters are explained in additional Metadata_parameters_7 spreadsheet.

Title: Supplementary Data 8.
Description: Connections between Nucleocytoviricota PolB genes and and 18S rRNA eukaryotic contigs.

Title: Supplementary Data 9.

Description: Inter- vs intra-taxonomic variation in functional genes distribution across Nucleocytoviricota MAGs and latitudes as assessed by generalized linear models. Variation was assessed as intra-taxonomic where the proportion of MAGs containing metacaspase changed within a phylogroup with latitude (p-value < 0.05), whereas inter-taxonomic variation was defined by a latitudinal gradient in the presence of phylogroups containing metacaspase, but the proportion of MAGs containing metacaspase within a phylogroup did not change with latitude (p-value > 0.05). Reported P values are from two-sided Wald tests. No adjustment for multiple comparisons was applied.

Title: Supplementary Data 10.
Description: Results of linear models predicting gross primary production (GPP) and Nucleocytoviricota richness (NCLDV_number) and relative abundance (NCLDV_rel_abund). For each predictor variable, we report its mean estimate ('Estimate'), estimated standard error ('Std. Error') and the statistical significance of the parameter estimate, along with model goodness of fit statistics.

Title: Supplementary Data 11.
Description: Coverage of Nucleocytoviricota functional genes in the study lakes. Functional gene annotation was performed by identifying KEGG Orthologs using KOfam Hidden Markov Model profiles (Aramaki et al. 2020) in anvi'o (Eren et al. 2015). Columns are lake name abbreviations corresponding to the "Lake" column in Supplementary Data 1. Cells represent coverage values for each of the genes in each lake.

Title: Supplementary Data 12.
Description: List of DNA polymerase B genes identified by GVClass HMM as matching to Nucleocytoviricota GVOGm0054. Parameters are explained in additional Metadata_parameters 12 spreadsheet.
