# Supplementary material for: The biogeography of giant viruses and interactions with Eukaryotes across European lakes
Source: Nat Commun. 2026 Sep 23;17:11056. doi: 10.1038/s41467-026-77765-w (PMC13691094; doi:10.1038/s41467-026-77765-w)
Supplement: Supplementary file 3 [file 41467_2026_77765_MOESM4_ESM.pdf]

nature portfolio

Corresponding author(s): Olesya Kolmakova

Last updated by author(s): Jul 19, 2026

Reporting Summary

Nature Portfolio wishes to improve the reproducibility of the work that we publish. This form provides structure for consistency and transparency in reporting. For further information on Nature Portfolio policies, see our Editorial Policies and the Editorial Policy Checklist.

Please do not complete any field with "not applicable" or n/a. Refer to the help text for what text to use if an item is not relevant to your study.
For final submission: please carefully check your responses for accuracy; you will not be able to make changes later.

Statistics

For all statistical analyses, confirm that the following items are present in the figure legend, table legend, main text, or Methods section.

n/a	Confirmed	
☐	☒	The exact sample size (n) for each experimental group/condition, given as a discrete number and unit of measurement
☐	☒	A statement on whether measurements were taken from distinct samples or whether the same sample was measured repeatedly
☐	☒	The statistical test(s) used AND whether they are one- or two-sided<br>*Only common tests should be described solely by name; describe more complex techniques in the Methods section.*
☐	☒	A description of all covariates tested
☐	☒	A description of any assumptions or corrections, such as tests of normality and adjustment for multiple comparisons
☐	☒	A full description of the statistical parameters including central tendency (e.g. means) or other basic estimates (e.g. regression coefficient) AND variation (e.g. standard deviation) or associated estimates of uncertainty (e.g. confidence intervals)
☐	☒	For null hypothesis testing, the test statistic (e.g. F, t, r) with confidence intervals, effect sizes, degrees of freedom and P value noted<br>*Give P values as exact values whenever suitable.*
☐	☒	For Bayesian analysis, information on the choice of priors and Markov chain Monte Carlo settings
☐	☒	For hierarchical and complex designs, identification of the appropriate level for tests and full reporting of outcomes
☐	☒	Estimates of effect sizes (e.g. Cohen's d, Pearson's r), indicating how they were calculated

Our web collection on statistics for biologists contains articles on many of the points above.

Software and code

Policy information about availability of computer code

Data collection	no software was used
Data analysis	Statistical analyses were undertaken with R version 4.4.1 (2024-06-14). DOC data were obtained using the Shimadzu TOC-control L software. Metagenomes were assembled using metaSpades v3.15.3 and binning performed with MetaBAT 2 v.1.7 using KBAse. GVClass v.1.1.1 was used to identify NCLDV MAGs in metagenomic bins. and characterize them NCLDV MAGs were refined and functionally annotated in anvi'o v.7.1. For NCLDV marker gene validation, we used GVClass v1.1.1, Prodigal v2.6.3, HMMER v3.4, and skani v.0.3.1. Phylogeny constructed with NSGTree v0.6.5 pipeline. The ssuextract pipeline v.0.9.0 was used for 18S rRNA gene extraction and annotation. Mapping was performed using BBMap v.35.85 and analyzed using CoverM v.0.6.1 and samtools v.1.16. Network interactions were inferred using FlashWeave v.1.6 BLAST+ 2.15.0 was used for nucleotide and amino acid sequence similarity search against the NCBI database [last accessed on 6 Feb 2024].

For manuscripts utilizing custom algorithms or software that are central to the research but not yet described in published literature, software must be made available to editors and reviewers. We strongly encourage code deposition in a community repository (e.g. GitHub). See the Nature Portfolio guidelines for submitting code & software for further information.

Data

Policy information about availability of data

All manuscripts must include a data availability statement. This statement should provide the following information, where applicable:

- Accession codes, unique identifiers, or web links for publicly available datasets
- A description of any restrictions on data availability
- For clinical datasets or third party data, please ensure that the statement adheres to our policy

Sequencing data used in this study have been deposited to ENA under project accession number PRJEB40644 [https://www.ebi.ac.uk/ena/browser/view/PRJEB40644]. Reference genomes of giant viruses are available here: [https://faylward.github.io/GVDB/]. All ecological data is available in Supplementary Data 1.

Research involving human participants, their data, or biological material

Policy information about studies with human participants or human data. See also policy information about sex, gender (identity/presentation), and sexual orientation and race, ethnicity and racism.

Reporting on sex and gender	NA
Reporting on race, ethnicity, or other socially relevant groupings	NA
Population characteristics	NA
Recruitment	NA
Ethics oversight	NA

Note that full information on the approval of the study protocol must also be provided in the manuscript.

Field-specific reporting

Please select the one below that is the best fit for your research. If you are not sure, read the appropriate sections before making your selection.

☐ Life sciences ☐ Behavioural & social sciences ☒ Ecological, evolutionary & environmental sciences

For a reference copy of the document with all sections, see nature.com/documents/nr-reporting-summary-flat.pdf

Ecological, evolutionary & environmental sciences study design

All studies must disclose on these points even when the disclosure is negative.

Study description	We investigated NCLDV MAGs across 87 European lakes, examining their biogeographic distribution, interactions with eukaryotes, and associations with local ecological conditions. Our findings also highlight the potential impacts of NCLDVs on freshwater ecosystems under changing climate conditions
Research sample	The sample consisted of surface water at the deepest point of each lake. We expected this location would be the best mixed and thus most representative of lake water quality
Sampling strategy	At each lake, we collected 10 L of water from the deepest point (or the point equidistant to all shorelines when no bathymetry information was available) in an acid-washed plastic carboy. We used this water to measure chemodiversity, biodiversity, and different physiochemical variables. We also separately measured CH4 and CO2 emissions from the lake surface using a floating chamber technique. Sample sizes were chosen based on maximizing the number of lakes that could be sampled at similar growing degree days in a single year.
Data collection	Ecosystem multifunctionality was measured in two main ways by a field team led by Jérémy Fonvielle and Andrew Tanentzap. Maximum primary production, total respiration, and bacterial protein production were measured by incubating lake water. Primary production and respiration were obtained from dissolved O2 measured using optical fibre sensors recording every second for ca. 5 minutes until reaching a plateau. Primary production was estimated as the difference in dissolved O2 between the start of the incubation and after exposure to a cold light lamp (5000K, 34,000 lux) for 3 hours. Respiration was the difference in dissolved O2 between the start and after 24 hours of dark incubation. Bacterial protein production was estimated based on 3H-leucine incorporation. 17nM of 3H-leucine was added to 1.5 mL of lake water and incubated in the dark for 1 hour. Following concentration of cells into a pellet, counts per minutes were measured with a portable liquid scintillation counter and 1 mL of HiSafe scintillation cocktail. For CH4 and CO2 emissions, these were measured with a floating chamber (0.07 m2 perimeter, 0.002 m3 area) connected to a Los Gatos Research ultraportable greenhouse gas analyser that uses off-axis integrated cavity output spectroscopy. After equilibrating the chamber, monitored the concentration of CH4 and CO2 inside the chamber every second for 15 mins. We estimated fluxes as the linear slope between the gas concentration and time. We also recorded water temperature and pH using a multiprobe (HI-99171, Hanna Instruments, USA) and measured water colour as the absorbance at 254nm using a spectrophotometer (Flame UV-

Vis, Ocean Optics, USA). Samuel Cottingham, Carolyn Ewins, Sophie Guillaume, Eleanor Sheridan, and Samuel Woodman helped record all these measurements.

Data on total nitrogen concentrations and dissolved organic carbon concentrations were collected by Jérémy Fonvielle, Andrew Tanentzap, and Yi Zhang on a Shimadzu TOC-TNM-L analyser.

Biodiversity data were collected by Jérémy Fonvielle with shotgun metagenomics sequencing. 1L of sample water was preserved in the field onto a 0.2 μm filter cartridge and all extractable DNA was sequenced for 50 base pair paired-end reads at a depth of ca. 10 million reads per sample by Novogene. Biodiversity data were collected by Jérémy Fonvielle with shotgun metagenomics sequencing. 1L of sample water was preserved in the field onto a 0.2 μm filter cartridge and all extractable DNA was sequenced for 50 base pair paired-end reads at a depth of ca. 10 million reads per sample by Novogene. Lucas Braga assembled and binned metagenomes from each lake using metaSPAdes and MetaBAT2. Frederik Schulz identified and taxonomically classified NCLDV MAGs with gvclass based on hallmark viral genes, followed by manual refinement in anvi'o by Olesya Kolmakova. Olesya Kolmakova and Yumary Vasquez annotated functional genes, and estimated their coverage from metagenomic read mapping. To capture additional viral markers missed by binning, Olesya Kolmakova and Yumary Vasquez screened assemblies for DNA polymerase B genes and validated their viral origin with BLAST-based pipelines. Frederik Schulz and Yumari Vasquez identified and annotated 18S rRNA genes from assembled contigs to represent eukaryotes, and estimated abundances of NCLDV MAGs and eukaryotic taxa from read mapping. Olesya Kolmakova and Yumary Vasquez inferred virus–host associations using co-abundance networks (FlashWeave) and further assessed for horizontal gene transfer through similarity searches.
Olesya Kolmakova tested how NCLDV taxonomic composition related to latitude, geography, and environmental variables using dissimilarity metrics, Mantel tests, and PERMANOVA. Andrew Tanentzap and Olesya Kolmakova assessed latitudinal trends in functional gene distributions with hurdle-Gamma models, distinguishing intra- versus inter-taxonomic drivers of variation.
Andrew Tanentzap evaluated ecosystem-level effects by modelling relationships between gross primary productivity, environmental factors, NCLDV richness and abundance.

Timing and spatial scale

We sampled approximately one lake per day from the 8 April 2019 to 24 September 2019. No sampling was performed between the 12 to 30 May 2019 and 25 July to 5 August to allow for staff downtime.

Data exclusions

Only lakes where sequencing depth allowed for NCLDV MAGs discovery were taken into analysis

Reproducibility

For lake water incubations to measure maximum primary production and total respiration, we used two replicates to confirm reproducibility, and three replicates for bacteria protein production. For CH_4 and CO_2 emissions, we repeated atmospheric equilibration and measurement twice, and a third time if the two fluxes differed by more than 10%.
For DOC measurements, we injected all samples at least three times in the machine, with up to five injection if the values differed by more than 10%.

For the genomic data, we recovered all species present in the positive control, thereby emphasizing that our protocol reproduced expected data. The libraries were quality controlled with a bioanalyzer to ensure the fragments were at the expected size and concentration.

Randomization

The order in which sites were sampled was designed to minimize seasonal variation in growing degree days.

Blinding

Blinding was not relevant in our study because we performed a field survey and not an experiment with treatments.

Did the study involve field work? ☒ Yes ☐ No

Field work, collection and transport

Field conditions

Field conditions were consistent with expected weather for the location and time of year.

Location

Latitude Longitude
39.55281667 16.02246667
40.93316667 15.60496667
42.12868333 12.24681667
42.60791667 11.94383333
43.07646667 17.4094
43.14788333 12.0673
43.865168 15.963112
43.882772 15.592607
44.887907 15.606738
45.68321667 10.73628333
45.86936667 6.161516667
45.95525 8.62777
46.36062 14.088032
46.42688333 6.609866667
46.58293333 14.15143333
46.604 14.58668333
46.6296 14.1255
46.672191 7.768283
46.6986 7.91395
46.79853333 6.6663

46.80805 6.303833333
47.00616667 8.3471
47.63855 13.77566667
48.02063333 11.10475
48.06878333 6.9533
48.69223333 6.9205
50.413287 7.271549
52.88853333 11.46245
53.155922 13.032579
53.347494 13.439851
53.45338333 15.06766667
53.51085 12.66841667
53.7912 12.10758333
54.12191667 10.41251667
56.31873333 15.3763
56.67526667 13.07176667
57.08735 12.51258333
57.77755 15.58343333
57.90081667 13.54426667
58.23513333 11.75205
58.61808333 14.48211667
58.86651667 17.38796667
58.99603333 11.53458333
59.25225 17.58803333
59.32255 12.34426667
59.611 12.301
59.73083333 12.75676667
59.840176 18.625799
59.9200365 15.4643798
60.14801667 16.34281667
60.15946667 15.74001667
60.188 10.71878333
60.4186979 11.2191583
60.5001265 22.2046013
61.14013333 28.49605
61.56633333 11.2144
61.66216667 16.58965
61.8771444 29.9168209
62.24396667 15.67738333
63.007 17.472
63.5406 29.12776667
63.8444 19.9467
64.05525 20.243167
64.215031 16.5330381
64.24178333 20.43035
64.26368333 16.42106667
64.88691667 28.92543333
65.52828333 21.41146667
65.58346667 17.48978333
65.5841 28.9106
65.78483333 18.1846
66.25448333 29.0401
66.31348333 18.13145
66.55463333 23.34941667
67.8158 20.55093333
68.43915 18.58283333
69.0161 20.85318333
69.14265 20.754
69.8178023 20.8624474
69.90976667 21.29786667
69.927 23.351
69.927736 23.352153
70.02411667 22.93045
70.0659 23.53928333
70.10895 23.56088333
70.803 25.748
71.1057
70.10895 23.56088333
70.803 25.748
71.1057 25.802683

Access & import/export

Sites were accessible and samples were collected in compliance with local, national, and international laws. There were no restrictions on import/export of samples to the UK for the purposes of our study.

All lakes were publicly accessible, and no permits were required for sample collection.

Disturbance

N/A. The study was non-destructive to the environment.

Reporting for specific materials, systems and methods

We require information from authors about some types of materials, experimental systems and methods used in many studies. Here, indicate whether each material, system or method listed is relevant to your study. If you are not sure if a list item applies to your research, read the appropriate section before selecting a response.

Materials & experimental systems

n/a	Involved in the study
☒	☐ Antibodies
☒	☐ Eukaryotic cell lines
☒	☐ Palaeontology and archaeology
☒	☐ Animals and other organisms
☒	☐ Clinical data
☒	☐ Dual use research of concern
☒	☐ Plants

Methods

n/a	Involved in the study
☒	☐ ChIP-seq
☒	☐ Flow cytometry
☒	☐ MRI-based neuroimaging

Plants

Seed stocks

NA

Novel plant genotypes

NA

Authentication

NA

This checklist template is licensed under a Creative Commons Attribution 4.0 International License, which permits use, sharing, adaptation, distribution and reproduction in any medium or format, as long as you give appropriate credit to the original author(s) and the source, provide a link to the Creative Commons license, and indicate if changes were made. The images or other third party material in this article are included in the article's Creative Commons license, unless indicated otherwise in a credit line to the material. If material is not included in the article's Creative Commons license and your intended use is not permitted by statutory regulation or exceeds the permitted use, you will need to obtain permission directly from the copyright holder. To view a copy of this license, visit http://creativecommons.org/licenses/by/4.0/
