# Supplementary material for: The biogeography of giant viruses and interactions with Eukaryotes across European lakes
Source: Nat Commun. 2026 Sep 23;17:11056. doi: 10.1038/s41467-026-77765-w (PMC13691094; doi:10.1038/s41467-026-77765-w)
Supplement: Supplementary file 4 [file 41467_2026_77765_MOESM5_ESM.pdf]

nature portfolio

Peer Review File

The biogeography of giant viruses and interactions with Eukaryotes across European lakes

Corresponding Author: Dr Olesya Kolmakova

This file contains all reviewer reports in order by version, followed by all author rebuttals in order by version.

Version 0:

Reviewer comments:

Reviewer #1

(Remarks to the Author)
General comments:
The study by Kolmakova investigated the diversity, distribution, and host association of NCLDVs in the surface layer of European lakes. They constructed 208 NCLDV environmental genomes (MAGs) from 87 lakes across a wide latitudinal gradient. The dataset is impressive in its spatial coverage. The study provides valuable findings, including novel virus-host associations and the impacts of UV and productivity on NCLDV diversity and function. However, I have several concerns, particularly regarding methodological or sampling limitations in addressing NCLDV diversity, which do affect the interpretation and conclusion of this study. In sum, while it is a valuable contribution and generally well written, I believe it does not represent a large enough advance and robustness for Nature Communications. Nevertheless, with methodological refinements, it could form the basis for an important contribution in freshwater viral ecology

Major issues:
The primary concern of this study is the low numbers (richness) of NCLDV MAGs from each lake. Thus, it is possible that the diversity information necessary for comparing lakes was not recovered in this study. The possible underestimation may be caused by MAGs being insufficiently constructed due to sequence depth or by the process of determining the presence of each MAG through mapping. In the aquatic ecosystems, several hundred to several thousand NCLDV species by a single marker gene (such as polB) and even tens to hundreds of species by MAGs can be yielded from a sample (Endo et al., 2020; 10.1038/s41559-020-01288-w; Meng et al., 2023, 10.1038/s41467-023-41910-6; Zhang et al., 2024, 10.1093/ismejo/wrae182). The values of this study fall significantly below those of these studies. The underestimation of MAG numbers affects not only diversity analyses but also network and AMG analyses, thereby affecting the conclusions of this paper. We recommend reconsidering the analytical procedures.

Specific issues:
L17: It has become common practice to use the phylum Nucleocitoviricota, which was formally accepted by ICTV, instead of NCLDV.
L99: Values of completeness and contamination would be helpful to further support the high quality of the MAGs.
L100: It is unclear what kind of diversity (Shannon, evenness, or richness) was compared.
L111: Based on the NCLDV frequency table (Table S3), most of the viral MAGs are sparsely distributed, and only 16 MAGs emerged at more than 10 sites. Such sparse data as well as low richness may decrease the specificity of a co-occurrence network (Berry and Widder, 2014, 10.3389/fmicb.2014.00219). The robustness of the network analysis should be carefully considered.
L116: It is generally quite difficult to classify mixotrophic and autotrophic protists based on a single marker gene like SSU rRNA, as the trophic mode can differ even in the same genus. Please add a detailed description of the classification method used.
L131-135: The relative NCLDV abundance would not be a good measure as it is strongly affected by the presence of other cellular organisms (including bacteria and multicellular organisms) and viruses. That is, the value will not simply represent the abundance of NCLDV. Comparing the ratio of an NCLDV marker (such as polB) to 18S rRNA frequency may be a more suitable indicator for assessing whether NCLDV was enriched relative to its host.
L141: The number of MAGs present in each lake looks very small. It might be due to the strict mapping cutoff criteria or to an insufficient number of constructed MAGs to map. The point here is that MAG-based analysis will not capture the true diversity of NCLDVs in each lake, thereby missing much information about the NCLDV genome and diversity.

Fig. 3: Surface temperature rather than latitude may be a more appropriate explanatory variable in this context. Higher latitudes do not necessarily show lower temperatures depending on the season and other factors.
L179: Although I am not a lake ecologist, I would like to know if the nutrient concentration truly decreases with latitude. The opposite trend is generally observed in the ocean. That is, high latitude has higher concentrations of inorganic nutrients than warm lower latitudes, because of the enhanced mixing of the water column. The authors need a more detailed explanation of the mechanisms underlying the nutrient dynamics in lake systems.
L187: The observed NCLDV richness is extremely low compared with a previous study in a lake system (Zhang et al., 2024, 10.1093/ismejo/wrae182). There is concern that, for some reason, the diversity of viruses in the environment may not be adequately recovered.
L198: I would suggest adding figures corresponding to this paragraph.
L209 and elsewhere: Some sentences in the Result section should be moved to the discussion.
L248: The effect of UV radiation on the NCLDV infection is somewhat questionable. I agree that UV can damage protists, but the harm of UV radiation to plankton primarily relates to the upper few metres of the water column, where biomass is relatively low; this does not appear to be a phenomenon that can be generalized to the entire lake.
L260: The relationship between lake depth and host diversity could be assessed by the present data.
L362: Did the author conduct dereplication of MAGs based on average nucleotide identity (ANI)? It would be an important process for obtaining a reliable abundance estimate.
L385: I suggest running the quality checks (completeness and contamination) for all the MAGs using CheckV. This information will help assess the reliability of the genome-scale analyses used in this study.
L432: I think it is appropriate to limit candidate hosts not only to those meeting the criteria, but also to the best-hit organisms for each query.

Reviewer #2

(Remarks to the Author)
This study systematically investigates the biogeographic distribution, virus-host interactions, and ecological effects of nucleocytoplasmic large DNA viruses (NCLDVs) in freshwater ecosystems through metagenomic sequencing of 87 European lakes. It fills a critical gap in the ecological research of NCLDVs in freshwater environments. The study design is rigorous with sampling covering a large latitudinal range (39°N-71°N). It identifies a large number of novel viral species and virus-host associations, and reveals the significant impact of NCLDVs on lake gross primary production (GPP), thus holding substantial scientific value. The most significant limitation of this manuscript is its near-complete omission of analyses linking the 208 recovered NCLDV genomes (including 188 putative new species) to viral evolution and taxonomy. While the authors generated a valuable genomic dataset—one of the largest for freshwater NCLDVs to date—they failed to leverage this resource to address foundational questions in virology: How do these new viruses relate to known NCLDV lineages evolutionarily? and Where do they fit in the global NCLDV taxonomic framework? This gap undermines the study’s potential to advance scientific understanding beyond a descriptive survey of viral diversity, as genomic, evolutionary, and taxonomic context are essential for interpreting the novelty, ecological relevance, and broader significance of the discovered viruses.

1.The authors generated 208 high-quality NCLDV metagenome-assembled genomes (MAGs), including 188 putative new species—a valuable genomic dataset. However, the manuscript fails to fully exploit these genomes to address key questions in virus taxonomy, ecology, and evolutionary biology, resulting in an understatement of the study’s broader significance.
（1）Below are some deficiencies in the description of phylogenetic analysis methods and results. Methods only states "A phylogenetic tree of NCLDV MAGs discovered in this study and from the reference database was built using IQ-TREE2" without specifying key parameters that directly impact tree accuracy, including: The set of marker genes or genomic regions used (e.g., whether the tree was built from concatenated core NCLDV marker genes like DNA polymerase B (PolB), topoisomerase II, or whole-genome alignments; if marker genes were used, how many and which specific GVOGs (Nucleocytoviricota orthologous groups) were selected). Supplementary Fig. 1 (the phylogenetic tree) and the main text provide no metrics to validate clade robustness, such as bootstrap support values (e.g., 1000 replicates) for key nodes, which indicate how consistently a clade is recovered across resampled datasets. In addition, the following key points should be taken into account when constructing the phylogenetic tree: Alignment and filtering procedures (e.g., whether sequences were aligned using MAFFT or MUSCLE; how ambiguous alignment regions were filtered, e.g., via Gblocks or trimAl; whether outgroups were included and which taxa were chosen as outgroups). Model selection for phylogenetic inference (e.g., whether the best-fit nucleotide/amino acid substitution model was selected via IQ-TREE2’s ModelFinder; if a specific model like LG+G or GTR+I+G was applied).
（2）The manuscript does not specify how "new species" were delimited (e.g., using average nucleotide identity [ANI], average amino acid identity [AAI], or digital DNA-DNA hybridization [dDDH]—standard metrics for viral taxonomy). Without these genomic thresholds, the claim of 188 new species is unsubstantiated (e.g., are MAGs with 95% ANI considered conspecific, or do they represent distinct species?). The authors mention that 22 of 32 Pimascovirales MAGs belong to the "ecologically uncharacterized family PM_01" but do not use their genomic data (e.g., core gene content, genome size, GC content) to characterize this family or propose new taxonomic ranks (e.g., families, genera) for other novel clades. The authors link NCLDV abundance/richness to environmental factors (e.g., latitude, SUVA) but do not use genomic traits (e.g., genome size, functional gene content, auxiliary metabolic genes [AMGs]) to explain why certain viruses thrive in specific environments.
2.The manuscript mentions "collecting duplicate samples from above the deepest point of each lake" but fails to specify the number of replicate samples (only "duplicate samples" is stated without clarifying if there are 3 or more biological replicates). It also does not provide information on the water depth during sampling or water stratification (e.g., whether sampling was conducted above the thermocline), which may affect the distribution of NCLDVs and their hosts. During filtration, "sampling stops when the filter clogs," but there is no description of the actual variation in filtration volume across samples from

different lakes, nor an explanation of whether this variation impacts the comparability of subsequent DNA extraction and sequencing data.
3.Virus-host associations are inferred only through co-occurrence analysis (FlashWeave) and horizontal gene transfer (HGT) signals. However, co-occurrence analysis may be driven by "shared habitats" rather than direct interactions, and HGT signals may also originate from indirect associations. For key newly discovered associations (e.g., the association between Mesomimiviridae and Ulvophycaea), attempt to verify the coexistence of viruses and hosts in samples via PCR, or supplement annotation results of host-specific functional genes (e.g., host metabolism-related genes) in viral genomes as supporting evidence.
4.The manuscript repeatedly references research results on marine NCLDVs but fails to systematically compare differences in biogeographic distribution, virus-host associations, and functional gene composition between freshwater and marine NCLDVs, as well as the underlying reasons.

Version 1:

Reviewer comments:

Reviewer #1

(Remarks to the Author)
I appreciate the authors for addressing my concerns, especially for checking the robustness of the diversity analysis.

Reviewer #2

(Remarks to the Author)
The author has well addressed most reviewer concerns. Nevertheless, Supplementary Fig. 1 should be moved to the main figures to better appeal to virologists. Further detailed explanations of this figure are also required in relevant parts of the main text.

Open Access This Peer Review File is licensed under a Creative Commons Attribution 4.0 International License, which permits use, sharing, adaptation, distribution and reproduction in any medium or format, as long as you give appropriate credit to the original author(s) and the source, provide a link to the Creative Commons license, and indicate if changes were made.
In cases where reviewers are anonymous, credit should be given to 'Anonymous Referee' and the source.
The images or other third party material in this Peer Review File are included in the article’s Creative Commons license, unless indicated otherwise in a credit line to the material. If material is not included in the article’s Creative Commons license and your intended use is not permitted by statutory regulation or exceeds the permitted use, you will need to obtain permission directly from the copyright holder.
To view a copy of this license, visit https://creativecommons.org/licenses/by/4.0/

Reviewer #1:
General comments:
The study by Kolmakova investigated the diversity, distribution, and host association of NCLDVs in the surface layer of European lakes. They constructed 208 NCLDV environmental genomes (MAGs) from 87 lakes across a wide latitudinal gradient. The dataset is impressive in its spatial coverage. The study provides valuable findings, including novel virus-host associations and the impacts of UV and productivity on NCLDV diversity and function. However, I have several concerns, particularly regarding methodological or sampling limitations in addressing NCLDV diversity, which do affect the interpretation and conclusion of this study. In sum, while it is a valuable contribution and generally well written, I believe it does not represent a large enough advance and robustness for Nature Communications. Nevertheless, with methodological refinements, it could form the basis for an important contribution in freshwater viral ecology.
We thank the Reviewer for appreciating the value, novelty, and effort involved in our study. We address their methodological concerns regarding NCLDV diversity below in response to their specific comments. We have further added new analyses, including of PolB markers, and clarified methods in ways that we hope increase the robustness and impact of our work.

Major issues:
The primary concern of this study is the low numbers (richness) of NCLDV MAGs from each lake. Thus, it is possible that the diversity information necessary for comparing lakes was not recovered in this study. The possible underestimation may be caused by MAGs being insufficiently constructed due to sequence depth or by the process of determining the presence of each MAG through mapping. In the aquatic ecosystems, several hundred to several thousand NCLDV species by a single marker gene (such as polB) and even tens to hundreds of species by MAGs can be yielded from a sample (Endo et al., 2020; 10.1038/s41559-020-01288-w; Meng et al., 2023, 10.1038/s41467-023-41910-6; Zhang et al., 2024, 10.1093/ismejo/wrae182). The values of this study fall significantly below those of these studies. The underestimation of MAG numbers affects not only diversity analyses but also network and AMG analyses, thereby affecting the conclusions of this paper. We recommend reconsidering the analytical procedures.
We agree that the limited sequencing depth inherent to genome-resolved approaches likely underestimates absolute viral richness, but our results are actually similar to others, including Meng et al. cited by the Reviewer as exemplary. Meng et al. analysed 1380 genomes that come from 928 samples (=1.49 genomes/sample). We recovered 208 genomes in 87 samples (=2.39 genomes/sample). Therefore, these differences between our studies and others are only partly due to sample size and may relate to habitat differences, i.e. comparing the global ocean vs lakes, rather than the mapping processing; though Meng used a slightly stricter breadth of coverage threshold. Nonetheless, we addressed this comment in two ways.

[#1] First, we ran the analysis exactly recommended by the Reviewer. We re-mined our dataset for PolB genes using updated HMM profiles from GVClass, recovering 1,498 regions matching GVOGm0054 (PolB) across our 87 metagenomes (Supplementary Table 12). This diversity is directly comparable to Endo et al. 2020, who detected 6818 PolBs in 370 metagenomes (18.4 per sample vs our 17.2 per sample). We then repeated the network analyses using this expanded marker-gene dataset (Supplementary Table 8). Despite the substantially higher PolB versus MAG richness, proportions of identified connections with different host types were unchanged. This concordance supports the robustness of our biogeographic conclusions. Importantly, while marker-gene approaches increase lineage

detection, they do not provide access to auxiliary metabolic genes (AMGs) or broader functional repertoires, which require genome-resolved data and are central to the functional analyses presented here. For this reason, we have retained the genome-resolved data in the main analyses and highlighted the concordance with PolB.

Added on lines 151-156 [line numbers refer to version with no track changes]: "To verify the robustness of our network analysis and account for potential undersampling inherent to the metagenomic approach, we repeated the network analysis using PolB (Supplementary Table 8) together with eukaryotic 18S rRNA genes. Networks were not significantly different in number of hosts identified (Fisher's simulated p-value = 0.114 based on 2000 replicates) (Supplementary Table 6; Supplementary Fig. 3), supporting the robustness of our conclusions."

Added on lines 531-537: "To assess patterns of host-giant virus co-occurrences further, we linked Nucleocytoviricota to eukaryotes using PolB and 18S rRNA sequences as markers for each group, respectively. For Nucleocytoviricota, we used GVClass to identify PolB sequences by utilizing HMMER and hmmsearch (-E 1e-10) with HMMs generated by GVClass (Supplemetary Table 12). We recovered 1,498 PolB sequences and confirmed that 1,460 PolB sequences were phylogenetically placed with Nucleocytoviricota genomes based on the phylogenetic nearest-neighbour identified with GVClass."

Added on lines 105-108: "These results were robust to potential under-sampling of MAGs because of unassembled genomes as the richness of the Nucleocytoviricota marker gene DNA polymerase B (PolB) strongly correlated with MAG richness across all lakes (Spearman rank correlation, ρ=0.76, p-value<0.001)."

[#2] Second, our primary objective was to investigate biogeography and functional patterns across lakes spanning a broad latitudinal gradient. For this reason, we used metagenomics to reconstruct NCLDV genomes and estimate diversity with one harmonised approach. Thus, while marker-gene-based approaches (e.g. PolB) like those used by Endo et al. (2020) are designed to maximize richness detection, and recover many more viral lineages than genome-resolved methods, they cannot answer functional questions. Likewise, Zhang et al. (2024) focused on a deeply sequenced individual system using assembly strategies designed to maximize viral genome recovery rather than the broad-scale patterns investigated here.

Importantly, all our analyses involve comparing differences between lakes, so our conclusions should not be compromised by potential underestimation of absolute richness. All samples were processed with identical workflows. Consequently, any underestimation of MAG numbers should be systematic across lakes rather than biased toward specific sites or regions. Under such standardized conditions, relative differences in community composition, functional gene repertoires, and network structure remain robust and informative, even if absolute richness is smaller than expected – which it is not, as explained above when comparing our smaller number of metagenome samples with past studies. However, we added the following to the start of the Discussion on lines 253-256 to acknowledge this point:

> "Although the absolute richness of MAGs was relatively low, it was comparable to other studies on a per-sample basis[3,5], and any underestimation would have been systematic across samples and thus preserve relative differences in community interactions and functional genes."

Specific issues:

L17: It has become common practice to use the phylum Nucleocitoviricota, which was formally accepted by ICTV, instead of NCLDV.

We have renamed all instances of NCLDV in the MS to Nucleocytoviricota, though we use the acronym informally here in our response.

L99: Values of completeness and contamination would be helpful to further support the high quality of the MAGs.

Values for completeness and contamination have been provided in Supplementary Table 4 using CheckV, as suggested by the Reviewer in their comment below on L385, and cited on line 105 of the Results. However, CheckV is poorly suited for NCLDVs because it was designed and benchmarked for smaller DNA viruses and bacteriophages. NCLDVs have very large, complex, and often highly divergent genomes, many of which are poorly represented in CheckV's reference database. Consequently, CheckV often underestimates genome completeness or incorrectly flags genuine viral sequences as contamination, making it unreliable for assessing NCLDV genome quality. There is no better alternative, to our knowledge, so we have caveated the Supplementary Table caption with these details.

L100: It is unclear what kind of diversity (Shannon, evenness, or richness) was compared.

Richness (number of NCLDV MAGs) was used. We replaced "diversity" with "richness".

L111: Based on the NCLDV frequency table (Table S3), most of the viral MAGs are sparsely distributed, and only 16 MAGs emerged at more than 10 sites. Such sparse data as well as low richness may decrease the specificity of a co-occurrence network (Berry and Widder, 2014, 10.3389/fmicb.2014.00219). The robustness of the network analysis should be carefully considered.

Thank you for raising this point. As explained in comment [#1] above, we repeated the analyses using PolB marker genes and number of identified host types remained consistent with our original results. The full changes to the text are given under comment [#1].

L116: It is generally quite difficult to classify mixotrophic and autotrophic protists based on a single marker gene like SSU rRNA, as the trophic mode can differ even in the same genus. Please add a detailed description of the classification method used.

We agree with the Reviewer. Added on lines 508-511: "Host trophic status was assigned based on the closest phylogenetic relative in a reference database[83] containing curated ecological and functional annotations of eukaryotic 18S rRNA sequences. When classification was uncertain, taxa were conservatively assigned as mixotrophic." Consequently, trophic categories should be interpreted as inferred ecological tendencies rather than definitive functional classifications.

L131-135: The relative NCLDV abundance would not be a good measure as it is strongly affected by the presence of other cellular organisms (including bacteria and multicellular organisms) and viruses. That is, the value will not simply represent the abundance of NCLDV. Comparing the ratio of an NCLDV marker (such as polB) to 18S rRNA frequency may be a more suitable indicator for assessing whether NCLDV was enriched relative to its host.

While it is true that metagenomic read counts are compositional and influenced by the presence of other taxa, this limitation applies universally to all metagenomic studies and does not invalidate ours uniquely. The number of NCLDV reads also did not depend on

sequencing depth, as stated on lines 123-124. This result indicates that NCLDV abundance does not simply reflect sampling bias / presence of other reads.

Nonetheless, we have also calculated the ratio of PolB to 18S reads in our samples suggested by the Reviewer. We found that this ratio was significantly positively correlated with the relative NCLDV abundance as now reported on lines 169-171. However, we have not replaced our analyses with this ratio because it can introduce other complexities and biases. For example, 18S rRNA gene copy number varies widely among Eukaryotes and its abundance depends on Eukaryotic composition as well as Eukaryotic biomass.

L141: The number of MAGs present in each lake looks very small. It might be due to the strict mapping cutoff criteria or to an insufficient number of constructed MAGs to map. The point here is that MAG-based analysis will not capture the true diversity of NCLDVs in each lake, thereby missing much information about the NCLDV genome and diversity.
We have addressed this point in our responses to the Reviewer's Major concern (see comments [#1] and [#2] above), so our responses are not repeated here.

Fig. 3: Surface temperature rather than latitude may be a more appropriate explanatory variable in this context. Higher latitudes do not necessarily show lower temperatures depending on the season and other factors.
We agree with the Reviewer that higher latitudes can be just as warm as lower latitudes at specific times. However, higher latitudes are colder, on average, at monthly and annual timescales. In this analysis, we test for adaptation to environmental conditions that occurs over these (and arguably even longer) timescales. So while a site in Sweden may be warmer than a site in Italy on a few given days, they will differ long-term and functional genes will be selected for this long-term signal rather than instantaneous surface temperatures at the exact time of our field sampling. These latter measurements may rather be more relevant for other "instantaneous" responses like GPP. We have clarified this point by changing lines 211-212 to the following: "This finding suggests that more favourable environmental conditions, such as <u>warmer long-term average temperatures</u>…"

This sentence is now more consistent with our initial predictions on lines 87-89, where we present latitude as a proxy that integrates long-term average temperature.

L179: Although I am not a lake ecologist, I would like to know if the nutrient concentration truly decreases with latitude. The opposite trend is generally observed in the ocean. That is, high latitude has higher concentrations of inorganic nutrients than warm lower latitudes, because of the enhanced mixing of the water column. The authors need a more detailed explanation of the mechanisms underlying the nutrient dynamics in lake systems.
Nitrogen and phosphorus concentrations are well known to decrease with latitude in lakes based on global comparative studies. For example, Abell et al. 2012 (<u>https://doi.org/10.1127/1863-9135/2012/0272</u>) and Yan et al. 2016 (<u>https://doi.org/10.1111/ele.12658</u>) demonstrate weak but statistically significant declines with increasing latitude in 1316 and 854 sites, respectively.

Unlike the ocean, where large-scale horizontal mixing can redistribute nutrients, lakes are isolated entities that strongly reflect the terrestrial catchments they drain. High-latitude catchments generally have lower soil P availability and weathering, reducing P export to lakes, as well as lower biological productivity that reduces N and P fluxes from soils and vegetation. Colder average temperatures at higher latitudes also reduce microbial

mineralization, slow sediment-water nutrient recycling, and have shorter ice-free seasons to accumulate nutrients in the water column. Finally, in the case of N, higher temperatures at low latitudes promote denitrification processes that removes available N from waters.

We have added the following to lines 211-216:
"This finding suggests that more favourable environmental conditions, such as warmer long-term average temperatures and higher nutrient availability, may select for phylogroups containing genes that enhance viral replication and host cell death, e.g. metacaspase-1 [28,29]. In our study lakes, total nitrogen concentrations declined with increasing latitude (ρ=-0.62, p-value<0.001), consistent with previously reported global patterns in lake nitrogen and phosphorus concentrations[30,31]."

Because the quoted line is the Results, we do not think it is appropriate to add a more detailed explanation of mechanisms underlying lake nutrient dynamics. We have now done so in the Discussion where we explain these results on lines 329-332:
"These associations between latitude and nutrients in lakes contrast with marine systems, where enhanced vertical mixing sustains high nutrient concentrations at high latitudes. In lakes, nutrient supply is primarily controlled by terrestrial catchments and climate that slow nutrient cycling at higher latitudes[53]."

L187: The observed NCLDV richness is extremely low compared with a previous study in a lake system (Zhang et al., 2024, 10.1093/ismejo/wrae182). There is concern that, for some reason, the diversity of viruses in the environment may not be adequately recovered.
We acknowledge that the observed NCLDV richness in our study is lower than that reported by Zhang et al. (2024). However, there are key differences in study design and objectives that explain this discrepancy. Our dataset includes a large number of lakes, providing a broad ecological snapshot across diverse environments, rather than aiming to recover exhaustively the full viral diversity in each system. In contrast, Zhang et al. focused on a single lake with deeper sequencing and combined long-read assemblies to maximize genome recovery. Importantly, as explained in our responses to the Reviewer's Major concern (see comments [#1] and [#2] above), the observed richness is comparable to others, and is sufficient both to detect the dominant NCLDV taxa and to compare viral community patterns across lakes. As reported in comment [#1], we added text to lines 253-256 that clarifies these points.

L198: I would suggest adding figures corresponding to this paragraph.
Added Supplementary Figure 4.

L209 and elsewhere: Some sentences in the Result section should be moved to the discussion.
We have moved this sentence to Discussion. We are happy to move other sentences that the Reviewer feels are out of place if they can provide us with more specific guidance.

L248: The effect of UV radiation on the NCLDV infection is somewhat questionable. I agree that UV can damage protists, but the harm of UV radiation to plankton primarily relates to the upper few metres of the water column, where biomass is relatively low; this does not appear to be a phenomenon that can be generalized to the entire lake.
We agree with the Reviewer. UV radiation will be harmful in the upper few meters of the water column, which is precisely where we sampled and where biomass peaks in lakes because light is abundant, temperatures are warm, and nutrients are available (especially after lake overturn). By contrast, the chlorophyll maximum in the oceans is typically much deeper

because it is primarily constrained by nutrients that regenerate from depth rather than by temperature gradients. We have clarified this point by adding the following on lines 288-290: "Any resulting damage could thus render protists in the epilimnion more susceptible to Nucleocytoviricota infections … and increase the richness and abundance of Nucleocytoviricota in our surface samples."

L260: The relationship between lake depth and host diversity could be assessed by the present data.
This comment is no longer relevant because we have reinterpreted our findings in the context of the literature, which suggest that depth may have an effect because of physical stability that promotes NCLDV persistence and accumulation.

Added on lines 298-302: "Third, lake depth was the strongest predictor of Nucleocytoviricota richness and abundance, potentially because deeper waters are more physically stable, thereby enabling Nucleocytoviricota to persist and accumulate on their hosts[47]. In support of this explanation, others have found greater compositional turnover over time for Nucleocytoviricota in epilimnetic waters than in the more stable hypolimnion[8]."

L362: Did the author conduct dereplication of MAGs based on average nucleotide identity (ANI)? It would be an important process for obtaining a reliable abundance estimate.
Added on lines 455-459: "The final set of 208 MAGs identified in this study was de-replicated and clustered by average nucleotide identity (ANI) alongside 1,384 Nucleocytoviricota reference genomes [24] with skani v0.3.1 [73]. Species-level clusters were defined using a 95% ANI threshold, following recommendations for species-rank virus operational taxonomic units [74] and a 70% aligned-fraction threshold."

L385: I suggest running the quality checks (completeness and contamination) for all the MAGs using CheckV. This information will help assess the reliability of the genome-scale analyses used in this study.
Done as explained in response to the Reviewer's comment about L99 above, so the full response and changes to the text are not repeated here.

L432: I think it is appropriate to limit candidate hosts not only to those meeting the criteria, but also to the best-hit organisms for each query.
Added on lines 528-530: "Only the highest-scoring matches were considered strong evidence for horizontal gene transfer, while all other hits were treated as weak evidence (Supplementary Table 6)."

Reviewer #2*:*
This study systematically investigates the biogeographic distribution, virus-host interactions, and ecological effects of nucleocytoplasmic large DNA viruses (NCLDVs) in freshwater ecosystems through metagenomic sequencing of 87 European lakes. It fills a critical gap in the ecological research of NCLDVs in freshwater environments. The study design is rigorous with sampling covering a large latitudinal range (39°N-71°N). It identifies a large number of novel viral species and virus-host associations, and reveals the significant impact of NCLDVs on lake gross primary production (GPP), thus holding substantial scientific value. The most significant limitation of this manuscript is its near-complete omission of analyses linking the

208 recovered NCLDV genomes (including 188 putative new species) to viral evolution and taxonomy. While the authors generated a valuable genomic dataset—one of the largest for freshwater NCLDVs to date—they failed to leverage this resource to address foundational questions in virology: How do these new viruses relate to known NCLDV lineages evolutionarily? and Where do they fit in the global NCLDV taxonomic framework? This gap undermines the study's potential to advance scientific understanding beyond a descriptive survey of viral diversity, as genomic, evolutionary, and taxonomic context are essential for interpreting the novelty, ecological relevance, and broader significance of the discovered viruses.

[#3] We thank the Reviewer for appreciating the ecological value and rigour of our study. Although foundational questions in virology were beyond the scope of our study, we have answered the Reviewer's specific questions by adding evolutionary and taxonomic context to the recovered NCLDV MAGs. We constructed phylogenies with previously known isolates and our MAGs, and interpret these results as now described on lines 108-117 *[line numbers refer to version with no track changes]*:

"To place the novel MAGs in an evolutionary and taxonomic context, we then constructed a phylogenetic tree including all 208 MAGs retrieved in this study alongside 1,383 representative reference genomes, enabling us to assign them to established Nucleocytoviricota lineages. The placement of our MAGs within multiple Nucleocytoviricota Orders, including deeply divergent lineages, indicates that our newly identified MAGs are related to known viruses and extend the diversity of established Orders into freshwaters (Supplementary Fig. 1). However, the recovery of distinct subclades comprised primarily of our MAGs, especially within the Imitervirales, suggests the potential for novel lower-rank lineages (i.e. within Orders) that may be affiliated uniquely with freshwaters."

This new text directly answers the Reviewer's question about how do viruses relate to known NCLDV lineages evolutionarily (i.e. they are interspersed within known lineages) and how they fit within the broader taxonomic framework (i.e. within established Orders but often as novel subclades suggesting new diversity at lower taxonomic ranks).

1.The authors generated 208 high-quality NCLDV metagenome-assembled genomes (MAGs), including 188 putative new species—a valuable genomic dataset. However, the manuscript fails to fully exploit these genomes to address key questions in virus taxonomy, ecology, and evolutionary biology, resulting in an understatement of the study's broader significance.

As explained above in response [#3], we have addressed the foundational questions that the Reviewer states are missing from the paper. The Reviewer also expands on this comment with two further points that we address immediately below.

(1) Below are some deficiencies in the description of phylogenetic analysis methods and results.

[#4] We have added text on lines 464-468 and 108-117 that describes these methods and results more clearly. Below we have also responded to each of the Reviewer's questions.

Methods only states "A phylogenetic tree of NCLDV MAGs discovered in this study and from the reference database was built using IQ-TREE2" without specifying key parameters that directly impact tree accuracy, including: The set of marker genes or genomic regions used (e.g., whether the tree was built from concatenated core NCLDV marker genes like DNA polymerase B (PolB), topoisomerase II, or whole-genome alignments; if marker genes were

used, how many and which specific GVOGs (Nucleocytoviricota orthologous groups) were selected).
We now state on lines 464-468 that we used whole-genome alignments with marker searches performed against the GVOG7 marker set consisting of superfamily II helicase, SFII (GVOGm0013); DNA-directed RNA polymerase subunit alpha/large RNA polymerase subunit, RNAPL (GVOGm0023); DNA polymerase family B, PolB (GVOGm0054); transcription initiation factor IIB, TFIIB (GVOGm0172); DNA topoisomerase II, TopoII (GVOGm0461); A32-like packaging ATPase, A32 (GVOGm0760); and virus late transcription factor 3, VLTF3 (GVOGm0890).

Supplementary Fig. 1 (the phylogenetic tree) and the main text provide no metrics to validate clade robustness, such as bootstrap support values (e.g., 1000 replicates) for key nodes, which indicate how consistently a clade is recovered across resampled datasets.
We have now added bootstrap support, as stated on lines 469-471 of the Methods. As there are too many leaves in the tree to display all support values, and most clades have high support (>90%), we only show highlight splits with medium to low support as explained in the revised figure caption.

In addition, the following key points should be taken into account when constructing the phylogenetic tree: Alignment and filtering procedures (e.g., whether sequences were aligned using MAFFT or MUSCLE; how ambiguous alignment regions were filtered, e.g., via Gblocks or trimAl; whether outgroups were included and which taxa were chosen as outgroups).
We aligned per-marker proteins with MAFFT and ambiguous alignments were trimmed with trimAl. The tree was rooted at the Pokkesviricetes group (Asfuvirales + Chitovirales). This information is now given on lines 464-466 and 758-760.

Model selection for phylogenetic inference (e.g., whether the best-fit nucleotide/amino acid substitution model was selected via IQ-TREE2's ModelFinder; if a specific model like LG+G or GTR+I+G was applied).
We now state on lines 471-473 that we used Q.pfam+R10+F model based on ModelFinder.

(2) The manuscript does not specify how "new species" were delimited (e.g., using average nucleotide identity [ANI], average amino acid identity [AAI], or digital DNA-DNA hybridization [dDDH]—standard metrics for viral taxonomy). Without these genomic thresholds, the claim of 188 new species is unsubstantiated (e.g., are MAGs with 95% ANI considered conspecific, or do they represent distinct species?).
We have now used standard thresholds of 95% ANI and a 70% aligned-fraction to identify putatively new species. Query MAGs forming clusters with no reference genome representatives were considered novel at the species level as now explained on lines 455-461, increasing our discovery from 188 to 205 new species.

The authors mention that 22 of 32 Pimascovirales MAGs belong to the "ecologically uncharacterized family PM_01" but do not use their genomic data (e.g., core gene content, genome size, GC content) to characterize this family or propose new taxonomic ranks (e.g., families, genera) for other novel clades.
We indeed examined core gene content, genome size, GC content (Supplementary Table 2), and functional annotations for this Order (data not shown). These analyses revealed gene repertoires consistent with those previously reported (e.g., Aylward et al. 2021) and did not

identify novel features that would meaningfully advance the central aims of this study. A detailed genomic or taxonomic revision of Pimascovirales is therefore beyond the scope of this MS and was not added to maintain focus.

The authors link NCLDV abundance/richness to environmental factors (e.g., latitude, SUVA) but do not use genomic traits (e.g., genome size, functional gene content, auxiliary metabolic genes [AMGs]) to explain why certain viruses thrive in specific environments.
We have examined the distribution and relative abundance of functional genes, including those associated with metabolic processes and stress responses, across the latitudinal gradient to assess why certain viruses thrive in specific environments as described on lines 186-198. We never related the distributions of individual viruses to SUVA. We only use SUVA to explain community patterns. We can certainly calculate community-weighted trait values (e.g. genome size and number of AMGs) and link those to environmental factors like latitude and elevation, but we worry that this analysis will complicate an already dense paper and dilute our focus on community patterns and ecosystem consequences.

2.The manuscript mentions "collecting duplicate samples from above the deepest point of each lake" but fails to specify the number of replicate samples (only "duplicate samples" is stated without clarifying if there are 3 or more biological replicates). It also does not provide information on the water depth during sampling or water stratification (e.g., whether sampling was conducted above the thermocline), which may affect the distribution of NCLDVs and their hosts.
Sampling was not conducted with replicates. As the spatial scale of the analysis was the individual lake, multiple replicates from the same lake would represent pseudoreplication rather than independent samples in our downstream ecological analyses. The "duplicate samples" were actually were used for a separate study and we apologise for the confusion that we caused. All samples were collected directly below the water surface to target near-surface viral communities, which are ecologically relevant for plankton-host interactions and most comparable across lakes of various depth and morphometry.

Changed on lines 379-385: "Surface water grab samples (0.5 m depth) were collected from April to September 2019 from 87 lakes located between 39°N to 71°N. A single sample was collected from above the deepest point of each lake (1 to 322 m) and 1L of water was immediately filtered through a Sterivex filter (polyethersulfone membrane, 0.22 um pore size, Millipore, USA). If the filter became clogged (ca. 5% of samples), we continued filtering with a new Sterivex until a total 1L of water was sampled and we sequenced the multiple filters and pooled reads downstream (see next section)."

During filtration, "sampling stops when the filter clogs," but there is no description of the actual variation in filtration volume across samples from different lakes, nor an explanation of whether this variation impacts the comparability of subsequent DNA extraction and sequencing data.
We apologise for this confusion. The original 2L figure was across both duplicate filters, which was misleading. 1L was filtered per filter. In about 5% of cases, filters clogged before reaching 1L and so we continued filtering on a new Sterivex unit. DNA was extracted from all Sterivex and, in the case of duplicates associated with clogging, we simply pooled reads post-hoc.

Changed on lines 380-385: "A single sample was collected from above the deepest point of each lake (1 to 322 m) and 1L of water was immediately filtered through a Sterivex filter

(polyethersulfone membrane, 0.22 um pore size, Millipore, USA). If the filter became clogged (ca. 5% of samples), we continued filtering with a new Sterivex until a total 1L of water was sampled and we sequenced the multiple filters and pooled reads downstream (see next section)."

3.Virus-host associations are inferred only through co-occurrence analysis (FlashWeave) and horizontal gene transfer (HGT) signals. However, co-occurrence analysis may be driven by "shared habitats" rather than direct interactions, and HGT signals may also originate from indirect associations. For key newly discovered associations (e.g., the association between Mesomimiviridae and Ulvophycaea), attempt to verify the coexistence of viruses and hosts in samples via PCR, or supplement annotation results of host-specific functional genes (e.g., host metabolism-related genes) in viral genomes as supporting evidence.

We thank the Reviewer for this suggestion. We now annotate host-related functional genes in viral genomes to supplement our analyses, exactly as the Reviewer recommends, using eggNOG-mapper.

Added on lines 135-142: "To support the Mesomimiviridae-Ulvophyceae association, we identified functionally relevant host genes in viral MAGs (Supplementary Table 7), including those involved in polyamine biosynthesis, suggesting modulation of host growth and viral replication; dTDP-L-rhamnose biosynthesis, potentially influencing cell surface structure; and deoxyribonucleotide and pyrimidine biosynthesis pathways that would support viral genome replication. Additionally, genes associated with glycosaminoglycan biosynthesis may contribute to host cell surface modification, facilitating viral attachment or spread."

We agree that PCR validation would be a valuable approach to confirm virus-host associations. However, this is far from trivial. We would potentially need to undertake thousands of PCR reactions, and in some cases, we no longer have DNA as it was entirely exhausted for the metagenomic sequencing libraries and other amplicon sequencing not presented in this study. Nonetheless, we have added text to acknowledge the potential for targeted PCR validation on lines 277-279: "Given that only a small fraction of Nucleocytoviricota genomes have been linked to confirmed hosts, most of the associations identified in our study require further validation, such as with PCR."

4.The manuscript repeatedly references research results on marine NCLDVs but fails to systematically compare differences in biogeographic distribution, virus-host associations, and functional gene composition between freshwater and marine NCLDVs, as well as the underlying reasons.

The scope of our manuscript is to investigate NCLDVs in freshwaters not to compare them to the oceans. We know very little about NCLDVs in freshwaters so we think that addressing this knowledge gap on its own has very high merit. We are unclear of what the specific biological questions might be that would necessitate a comparison between freshwater and marine NCLDVs. That said, we acknowledge that most research on NCLDVs in environmental contexts has occurred in marine environments and we have expanded interpretation of biogeographic distributions, virus-host associations, and functional gene composition in the context of what is known from marine studies. We think that doing so strikes the appropriate balance between the scope of our current study and contextualising our results within existing literature.

Added on lines 336-358: "Much of our understanding about the host associations, biogeography, and functional gene composition of Nucleocytoviricota communities is derived

from the oceans, where environmental filters and dispersal processes shape distinct patterns from freshwaters. For example, lakes receive proportionally greater inputs of terrestrial organic matter than marine environments, especially the open ocean[54], resulting in chemically different substrates for eukaryotic consumers. Consequently, lakes may offer different ecological niches for hosts that influence Nucleocytoviricota distributions, and we detected frequent associations between Nucleocytoviricota and both zooplankton and fungi that are less apparent in the oceans[54]. Lakes also differ from marine environments, especially the open ocean, because weather-driven mixing can rapidly redistribute organisms across depths and restructure encounter rates between viruses and their hosts in contrast to the more persistent circulation patterns of oceans[1]. This mechanism is consistent with our observation that Nucleocytoviricota richness and abundance were better explained by lake-specific characteristics, such as depth and organic matter quality, rather than by latitude alone. In marine systems, Nucleocytoviricota composition is strongly structured by basin and biome because it tracks major water masses and distributions of primarily photosynthetic planktonic eukaryotes, resulting in large-scale biogeographic patterns[5]. By contrast, the discrete catchment boundaries of our study lakes may impose stronger dispersal limitation and compositional differences over small spatial scales, as reflected by the positive correlation between Nucleocytoviricota compositional dissimilarity and geographic distance. Finally, while marine Nucleocytoviricota commonly encode AMGs involved in metabolism and stress responses that align with broad oceanographic gradients[55], our results suggest that their distribution in lakes primarily reflects local environmental conditions, such as nutrients, light availability, and thermal structure."

REVIEWERS' COMMENTS

Reviewer #1 (Remarks to the Author):

I appreciate the authors for addressing my concerns, especially for checking the robustness of the diversity analysis.

We thank the reviewer for their positive feedback and are pleased that our additional analyses have addressed their concerns.

Reviewer #2 (Remarks to the Author):

The author has well addressed most reviewer concerns. Nevertheless, Supplementary Fig. 1 should be moved to the main figures to better appeal to virologists. Further detailed explanations of this figure are also required in relevant parts of the main text.

We thank the reviewer for this helpful suggestion. As recommended, we have moved Supplementary Fig. 1 to the main figures (now Fig. 1) to improve its visibility and accessibility to readers. The phylogenetic tree is already described in several parts of the main text, and we consider the existing explanation sufficient for its interpretation.
